# Repurposing of FDA Approved Drugs Against SARS-CoV-2 Papain-Like Protease: Computational, Biochemical, and *in vitro* Studies

**DOI:** 10.3389/fmicb.2022.877813

**Published:** 2022-05-10

**Authors:** Rajkumar Kulandaisamy, Tushar Kushwaha, Anu Dalal, Vikas Kumar, Deepa Singh, Kamal Baswal, Pratibha Sharma, Kokkula Praneeth, Pankaj Jorwal, Sarala R. Kayampeta, Tamanna Sharma, Srinivas Maddur, Manoj Kumar, Saroj Kumar, Aparoy Polamarasetty, Aekagra Singh, Deepak Sehgal, Shivajirao L. Gholap, Mohan B. Appaiahgari, Madhumohan R. Katika, Krishna K. Inampudi

**Affiliations:** ^1^Department of Biophysics, All India Institute of Medical Sciences, New Delhi, India; ^2^Department of Chemistry, Indian Institute of Technology-Delhi, New Delhi, India; ^3^Department of Neurosurgery, All India Institute of Medical Sciences, New Delhi, India; ^4^Department of Medicine, All India Institute of Medical Sciences, New Delhi, India; ^5^Research and Development Division, Srikara Biologicals Private Limited, Tirupati, India; ^6^Central Research Laboratory Mobile Virology Research and Development BSL3 Lab, Employees' State Insurance Corporation Medical College and Hospital, Hyderabad, India; ^7^Faculty of Biology, Indian Institute of Petroleum and Energy, Visakhapatnam, India; ^8^Virology Lab, Department of Life Sciences, Shiv Nadar University, Greater Noida, India; ^9^Stem Cell Facility and Regenerative Medicine, Nizam's Institute of Medical Sciences, Hyderabad, India

**Keywords:** COVID-19, SCoV-2, Papain-like protease, FDA drug, virtual screening, enzymatic assays, HCoV-229E, antiviral assays

## Abstract

The pandemic caused by SARS-CoV-2 (SCoV-2) has impacted the world in many ways and the virus continues to evolve and produce novel variants with the ability to cause frequent global outbreaks. Although the advent of the vaccines abated the global burden, they were not effective against all the variants of SCoV-2. This trend warrants shifting the focus on the development of small molecules targeting the crucial proteins of the viral replication machinery as effective therapeutic solutions. The PLpro is a crucial enzyme having multiple roles during the viral life cycle and is a well-established drug target. In this study, we identified 12 potential inhibitors of PLpro through virtual screening of the FDA-approved drug library. Docking and molecular dynamics simulation studies suggested that these molecules bind to the PLpro through multiple interactions. Further, IC_50_ values obtained from enzyme-inhibition assays affirm the stronger affinities of the identified molecules for the PLpro. Also, we demonstrated high structural conservation in the catalytic site of PLpro between SCoV-2 and Human Coronavirus 229E (HCoV-229E) through molecular modelling studies. Based on these similarities in PLpro structures and the resemblance in various signalling pathways for the two viruses, we propose that HCoV-229E is a suitable surrogate for SCoV-2 in drug-discovery studies. Validating our hypothesis, Mefloquine, which was effective against HCoV-229E, was found to be effective against SCoV-2 as well in cell-based assays. Overall, the present study demonstrated Mefloquine as a potential inhibitor of SCoV-2 PLpro and its antiviral activity against SCoV-2. Corroborating our findings, based on the *in vitro* virus inhibition assays, a recent study reported a prophylactic role for Mefloquine against SCoV-2. Accordingly, Mefloquine may further be investigated for its potential as a drug candidate for the treatment of COVID.

## Introduction

Severe acute respiratory syndrome coronavirus 2 (SARS-CoV-2/SCoV-2), a member of human beta coronaviruses, is responsible for the ongoing COVID pandemic and is accountable for high morbidity and mortality. Infections with SCoV-2 have been majorly asymptomatic with about 15% of infections resulting in symptomatic clinical cases, and a minor percentage succumbing to the disease. To this date, the world has recorded about 3.7 billion clinical cases and 5.7 million deaths, and the occurrence of clinical cases continues to rise due to frequent outbreaks caused by new variants of SCoV-2. Besides, the pandemic has impacted the world socially and economically. Compared to SCoV-1 and MERS-CoV, the COVID-19 pandemic has demonstrated high transmissibility and therefore remains highly challenging to contain.

The genome of SCoV-2 is an ~30 kb long single-stranded positive sense RNA molecule. The viral genome codes for 4 structural proteins and 16 non-structural proteins (nsp1 to nsp16), which include two viral proteases, Papain-like protease (PLpro/nsp3) and the 3-chymotrypsin-like protease (3CLpro/nsp5). These proteases help in the processing of the polyprotein into functional proteins. Similarly, the genome also codes for proteins of the viral replicase complex consisting of RNA-dependent RNA polymerase (RdRp/nsp12) and other auxiliary proteins that are essential in viral replication (Anand et al., 2003; Harcourt et al., 2004). Interfering with any of these crucial protein functions can affect the viral replication, thereby the production of viral progeny and finally the disease outcome. The SCoV-2 PLpro recognizes the LXGG motifs located at the junctions of nsp1/2, nsp2/3, and nsp3/4 in the polyprotein and hydrolyses them to produce functionally active proteins. Another crucial function of the PLpro is the cleavage of host ubiquitin and ISG15 (regulators of host innate immune pathways), which trigger host immune responses, and thereby helps the virus to spread effectively inside the infected host (Devaraj et al., 2007; Klemm et al., 2020; Maiti, 2020; Shin et al., 2020). Due to its multifunctional and indispensable roles during the viral life cycle, PLpro has become a crucial target for anti-SCoV-2 drug discovery.

The medical emergency due to the COVID pandemic has prompted researchers from various disciplines across the globe to prioritise their research focus in quickly understanding various aspects of SCoV-2 biology. Accordingly, there is an abundance of experimental data available on the biochemical and structural aspects of SCoV-2 PLpro that can be very handy in finding potential antivirals and/or designing novel molecules targeting this important viral component. The SCoV-2 PLpro shares 83% similarity with SCoV-1 PLpro, while it shares 31% and 53% similarity with the MERS-CoV and HCoV-229E, respectively (Huang et al., 2020; Lu et al., 2020). The PLpro consists of four domains: N-terminal Ubiquitin like domain (Ub1), thumb domain, finger domain, and the palm domain. The catalytic triad contains the Cys111, His272, and Asp286, which are crucial in polyprotein processing. The catalytic site has a zinc binding site, which plays a crucial role in dictating the enzymatic activity of PLpro. The zinc binds to the four cysteine residues (Cys_189_, Cys_192_, Cys_224_, and Cys_226_) in a tetrahedral arrangement and is responsible for facilitating the structural integrity and correct protein folding. Due to this reason, the zinc binding site has become a druggable site using Zn-ejector-based drugs (Sargsyan et al., 2020a,b).

Recent drug-discovery research targeting the SCoV-2 PLpro proposed GRL0617 and its selected analogues as potential inhibitors of viral protease *in vitro* and inhibitors of SCoV-2 replication in cell-based assays (Ratia et al., 2008; Freitas et al., 2020; Rut et al., 2020; Shin et al., 2020; Fu et al., 2021; Osipiuk et al., 2021). Similarly, Delre et al. performed *in silico* screening of phase III and IV tested and clinically approved drugs against SCoV-2 PLpro, and proposed a total of 24 drugs (22 non-covalent and 2 covalent) as promising inhibitors of SCoV-2 PLpro (Delre et al., 2020). While majority of the drugs proposed in the recent reports are yet to be tested in preclinical and clinical studies, currently there is no specific clinically approved effective therapy to contain the ongoing pandemic and hence, there is a need to identify/design novel antivirals against SCoV-2. In this study, virtual screening of FDA-approved drug library (for repurposing) against SCoV-2 PLpro has been employed to identify molecules potentially capable of inhibiting SCoV-2 replication. The retrospective analysis of per-residue decomposition of the binding free energy calculations revealed the role of individual residues in the inhibition by selected molecules. Next, inhibitory activity of the identified molecules using GRL0617, as a known reference against SCoV-2 PLpro, was demonstrated biochemically by PLpro enzyme assays. Here, we found that FAD and Mefloquine have IC_50_ values in the micromolar range similar to GRL0617. The molecules, Glutathione disulphide, Lopinavir, Darunavir, and Ritonavir exhibited moderate IC_50_ values against SCoV-2 PLpro. Further, through cell-based antiviral assays, we have demonstrated superior antiviral activities for Lopinavir and Mefloquine against HCoV-229E, which was used as a surrogate for SCoV-2 (Ma et al., 2020; Butot et al., 2021; Owen et al., 2021; Pasquereau et al., 2021). We further validated the antiviral activity of Mefloquine against SCoV-2 in Vero E6 cells. Further validating the anti-SCoV-2 activity of Mefloquine, a recent study by Shionoya et al. proposed a prophylactic role for Mefloquine based on the findings *in vitro* in SCoV-2-infected Vero E6 cells (Shionoya et al., 2021). Therefore, we propose Mefloquine as a SCoV-2 PLpro inhibitor for further development in preclinical and clinical studies for drug repurposing.

## Materials and Methods

### *In silico* Screening of FDA-Approved Drugs Against SCoV-2 PLpro

#### PLpro Protein Receptor and Ligand Preparation

The 3D-coordinates of SCoV-2 PLpro were obtained from the protein database (PDB ID:6WX4; Rut et al., 2020) for *in silico* studies. The PLpro structure with the Zn^2+^ atom using the Maestro v1 protein preparation wizard (Friesner et al., 2004) at the physiological pH 7.4 and by allowing modeling of missing loop residues. All the water molecules were removed from the PLpro protein followed by the addition of hydrogen atoms. The optimised protein was subjected to energy minimization using the liquid simulation (OPLS-3) force fields for all atoms in the structure. The receptor grid box was generated using Glide v7.1 at the centre of the active site residues (Trp93, Trp106, Asp108, Asn110, Cys111, His272, Asp286; Ratia et al., 2006) of the PLpro protein. The library of FDA-approved drugs was retrieved from Drug bank v5.0 in the SDF file format. The retrieved ligands were prepared using the Ligprep module for ligand preparation (Friesner et al., 2004). Hydrogen atoms were added to the ligand library followed by energy minimization using the OPLS-3 force field to obtain low energy molecules for virtual screening (Friesner et al., 2004).

#### Virtual Screening

High-throughput Virtual Screening (HTVS) was carried out by molecular docking analysis using Glide v7.1 and employing the defined receptor grid against the set of prepared ligand libraries. A total of 2,467 FDA-approved drugs from the Drug bank database were screened against SCoV-2 PLpro in HTVS mode and the top 10% hits were further docked using the Standard Precision (SP) mode. Further, the top 10% ligands were selected and subjected to docking with Extra Precision (XP) mode in Glide. The best 12 (Rut et al., 2020) docked ligands were selected for MD simulations based on the glide energy, docking score, and the interactions with active site residues of PLpro protein for further refinement (Table 1).

**Table 1:** List of FDA approved drugs screened against SARS-CoV-2 PLpro by *in silico* screening.

**S. No**.	**Database ID**	**Generic name**	**2D structures**	**Docking score**	**Glide energy**	**H-bond interactions**	**Ionic interactions**	**Hydrophobic interactions**
1.	DB00284	Acarbose	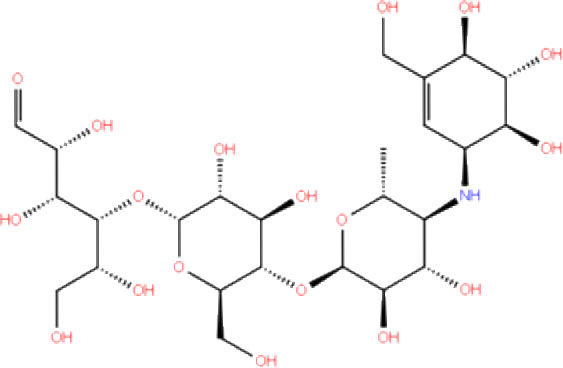	−11.578	−52.918	Asn109, Trp106, His272, Ala 288	-	-
2.	DB03310	Glutathione Disulphide	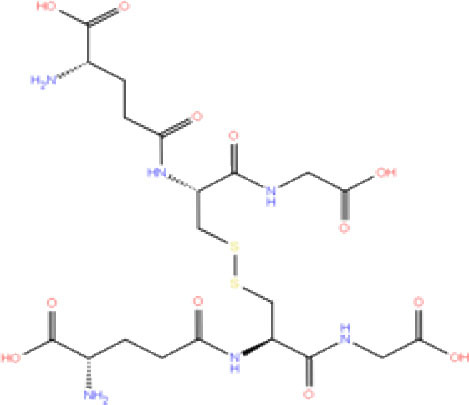	−8.143	−50.076	Trp106, Cys111, His272, Thr265, Lys274, Asp286, Ala 288	His272, Lys274	Trp106
3.	DB06796	Mangafodipir	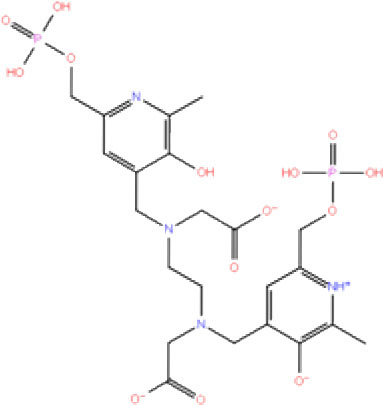	−9.656	−70.955	Ile104, Asn109, Asn110, Thr265, His 272	His272, Lys274, Asp286	Ala106, His 272 (Pi-stacking)
4.	DB03147	FAD	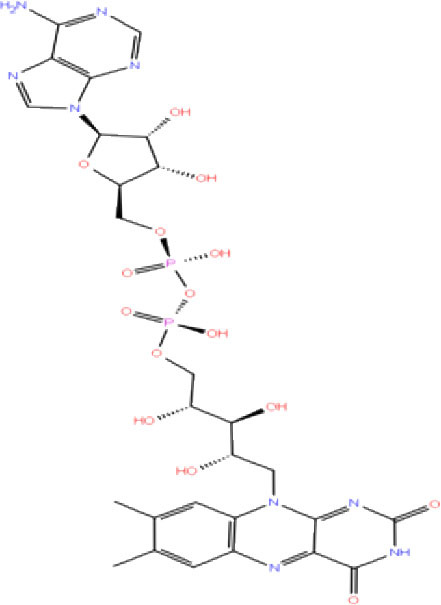	−9.836	−68.170	Lys92, Lys94, Trp 106, Asp108, Asp286, Ala288	-	-
5.	DB09156	Iopromide	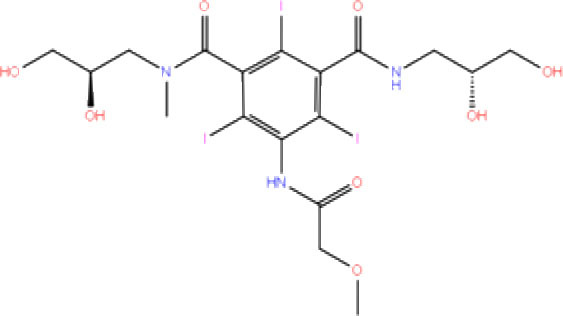	−8.306	−55.399	Trp106, Asn109, Asn110, Thr265, His272, Asp286	-	His272 (Pi-stacking)
6.	DB01698	Rutin	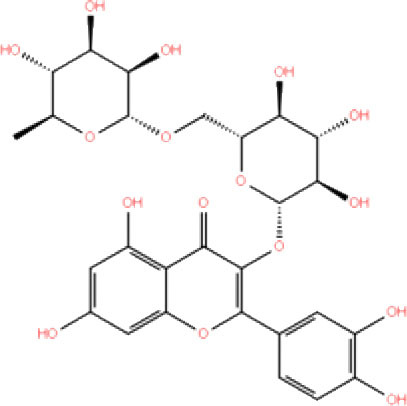	−8.604	−50.725	Trp106, Asn109, His272, Gly 271, Asp286, Ala288	-	Trp106, His272
7.	DB012434	Steviolbioside	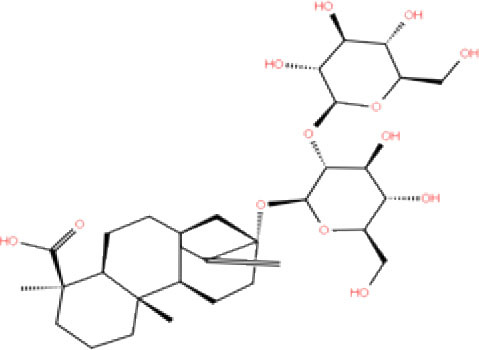	−8.289	−43.118	Trp106, Cys270, Gly271	-	Trp106
8.	DB012942	Lactitol	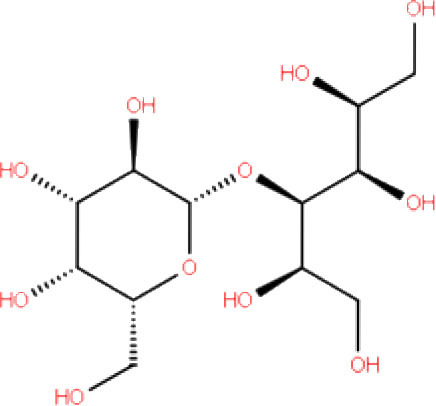	−7.995	−33.789	Trp106, Asn109, Cys270, Gly271, His272,	-	-
9.	DB01601	Lopinavir	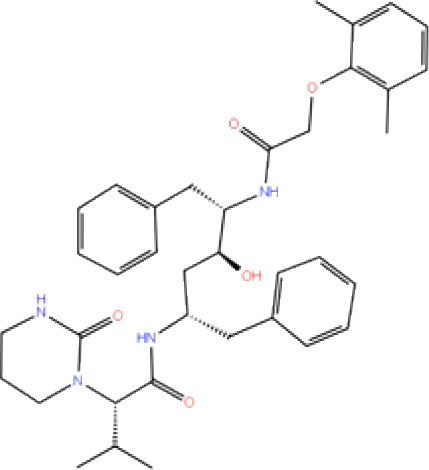	−2.882	−45.604	Trp106, Asp286, Ala288	-	Trp106
10.	DB00358	Mefloquine	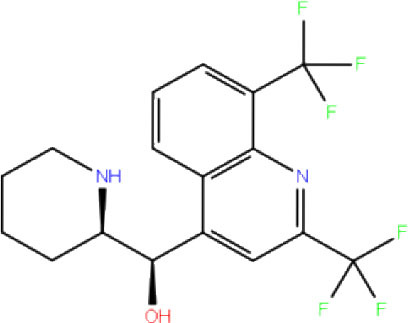	−1.48	−27.074	Trp106, Asp286, Ala288	-	Trp106
11.	DB01264	Darunavir	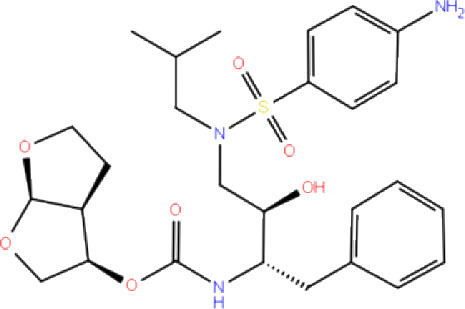	−3.261	−39.824	Trp106	-	Trp106 (Two hydrophobic and one Pi-stacking)
12.	DB00503	Ritonavir	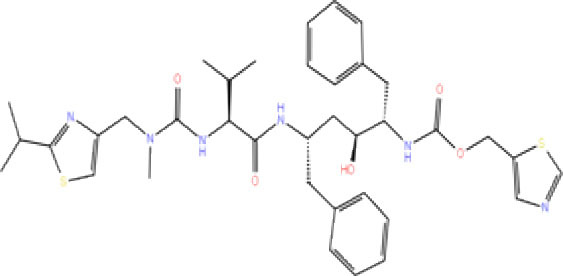	−4.151	−49.703	Lys274	-	Trp108, Ala114

#### Molecular Dynamics (MD) Simulations

The binding stability of the selected hits obtained from screening was assessed by MD simulations of their docked complexes using GROMACS software suite (version 2018.7). The simulations were performed using GROMOS 54A7 force field parameters for protein atoms and the PRODRG server was used for building topological parameters of the ligand molecules since it also included chemical scaffolds which were similar to our ligands (Schüttelkopf, 2004). Each complex was solvated in a dodecahedron box with a minimum 1 nm distance from protein atoms using Simple Point-Charge (SPC) water model. Solvated complexes were neutralised with Cl^−^ ions and energy minimised using the steepest descent algorithm until maximum force was <1,000 kJmol^−1^ nm^−1^. The energy minimised electroneutral system was equilibrated under a canonical ensemble (NVT) followed by an isothermal-isobaric (NPT) ensemble for 100 ps each. The temperature and pressure of the system were maintained at 300 K and 1 bar, respectively, using a modified Berendsen thermostat and Parrinello-Rahman barostat. Bond lengths were constrained using the P-LINCS algorithm while Particle Mesh Ewald (PME) was used for handling the long-range component of the non-bonded interactions with standard parameters. The Leapfrog algorithm was used to integrate Newton's equation of motion with a step size of 2 fs. The production simulation was performed for a time duration of 100 ns for all the 12 complexes along with unliganded protein. Position restraints were applied to zinc atoms during the simulation. Periodic boundary conditions were considered in all three dimensions throughout. The output trajectory was analysed using GROMACS modules and in-house python scripts.

#### Free Energy Estimation Using MMPBSA

The binding interaction was quantified by estimation of binding free energy for each of the MD refined complexes using Molecular Mechanics Poisson–Boltzmann Surface Area (MM-PBSA) method employing the g_mmpbsa tool (Kumari and Kumar, 2014). The g_mmpbsa uses Assisted Poisson Boltzmann Solver (APBS) program for computing polar solvation energy and Solvent Accessible Surface Area (SASA) model for polar solvation energy (Miller et al., 2012). MM-PBSA calculations were performed for the last 25 ns of the simulation trajectory after convergence (Chaudhary and Aparoy, 2020). Based on MM-PBSA calculations and interaction profile, nine molecules were selected for inhibition assays and were procured from Sigma. Further, in a retrospective analysis, per-residue decomposition of the binding free energy was done to dissect the role of individual residues in inhibition of Mefloquine and Lopinavir.

#### HCoV-229E PLpro Homology Modelling

The HCoV-229E PLpro protein sequence was retrieved from previously reported sources (Loyola eCommons, 2014; Ye et al., 2021). The three-dimensional structure of HCoV-229E PLpro was modelled using I-TASSER followed by refinement using GalaxyRefine web server (Heo et al., 2013). Molprobity server (Williams et al., 2018) was used for validation of the modelled HCoV-229E PLpro and was subsequently used to dock all selected molecules for comparative studies with SCoV-2 PLpro.

#### Computational Analysis of Signalling Pathways Between SCoV-2 and HCoV-229E

To explore HCoV-229E as a surrogate virus for drug-discovery studies (Ma et al., 2020; Butot et al., 2021; Owen et al., 2021; Pasquereau et al., 2021) in a BSL-3 laboratory, we compared the similarities and dissimilarities of signalling pathways involved in disease pathogenesis between HCoV-229E and SCoV-2. Genomic sequences of HCoV-229E (NC_002645) and SCoV-2 (NC_045512) were retrieved from the National Center for Biotechnology Information (NCBI) and aligned using Geneious software to find a similarity between the two genomes.

### Cloning, Expression, and Purification of PLpro Protein in a Bacterial Expression System

**Figure 1:**
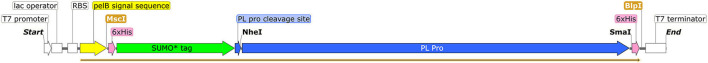
Depicts the strategy used to clone the cDNA coding for SARS-CoV-2 PLpro in pET22b vector system for bacterial expression. The PLpro gene codons were optimized, synthesized, and subcloned into the pET22b vector with MscI and BIpI restriction site. N-terminal end of the clone having a 6X His tag followed by SUMO tag consisting of PLpro autocleavage site. On the other hand, C-terminals end up having 6x His tag for purification. *Autocleavable.

The cDNA sequence coding for SCoV-2 PLpro was codon-optimised for bacterial expression and was custom-synthesised in pET22b vector backbone by GeneScript, USA. The expression cassette consists of the N-terminal 6X-His tag followed by SUMO tag with PLpro cleavage site for autocleavage during protein expression and a 2nd 6x His tag at the C-terminus for purification as shown in Figure 1 and Supplementary Information 8. The PLpro plasmid construct was transformed into C43 cells and the recombinant bacterial clones grown at 18°C were induced with 1 mM ZnCl_2_ and 0.5 mM IPTG to allow overexpression of recombinant PLpro. Expression of the recombinant PLpro was confirmed by SDS-PAGE analysis as well as by immunoblot analysis using anti-his antibody using standard methods. For large scale production and purification of the recombinant PLpro, selected recombinant bacterial clone grown in 2x YT broth broth was induced at OD_600_~0.6 under previously optimised conditions. Induced bacterial culture was harvested by centrifugation, cell pellet was lysed by sonication in the lysis buffer (50 mM Tris-HCl, 150 mM NaCl, 10 mM Imidazole,.1% Triton X-100, and 2 mM BME, pH 8.5) and then centrifuged at 20,000 × g for 1 h to obtain a clear supernatant. The supernatant was passed through pre-equilibrated HisTrap FF column (5 ml, Cytiva) with the lysis buffer and the bound protein was eluted (50 mM Tris-HCl, 150 mM NaCl, 250 mM Imidazole, and 2 mM BME, pH 8.5). The eluted PLpro protein fractions were further purified by size-exclusion chromatography using HiLoad 26/600 Superdex 200 pg column equilibrated with storage buffer (50 mM Tris, 150 mM NaCl, 2 mM DTT pH 8.5). The purified fractions were concentrated using 10 kDa molecular cut-off Amicon tube (Amicon Ultra, Merck Millipore) and stored in a buffer containing 20% glycerol at −80°C for further experiments.

### Optimization of SCoV-2 PLpro Assay

The fluorescence-based enzymatic activity of PLpro was analysed using the peptide-AMC substrate (Z-Arg-Leu-Arg-Gly-Gly-AMC, Cat. # 79997, Bachem Bioscience, USA) in a 96-well plate. In principle, PLpro recognizes the consensus cleavage motif, LXGG (X = any amino acid, L = Leucine, and G = Glycine), in the peptide substrate and hydrolyses it to allow fluorescence emission (excitation λ: 380 nm; emission λ: 460 nm). In order to determine the optimal enzyme concentration, 25 μM PLpro-substrate was incubated with increasing concentrations of recombinant PLpro (0-10 μM) in the assay buffer (20 mM Tris pH 8.5, 100 mM NaCl, and 2 mM DTT) at 37°C for 45 mins in a 50 μL reaction volume. The fluorescence emission was measured at 460 nm after excitation at 360 nm using the Tecan/Spectramax multimode plate reader. Similarly, to determine the K_m_ of the substrate, assays were set up using increasing concentrations (0-150 μM) of substrate in the presence of optimum concentration of the recombinant SCoV-2 PLpro at 37°C for 45 min and the fluorescence signals were recorded. Initial velocities of AMC release were normalized to a standard curve and the velocity vs. substrate concentration plot was further analysed by Michaelis-Menten equation (Sargsyan et al., 2020b; Fu et al., 2021; Osipiuk et al., 2021; Zhao et al., 2021)_._

### Synthesis of GRL0617 Reference Drug

**Figure 2:**
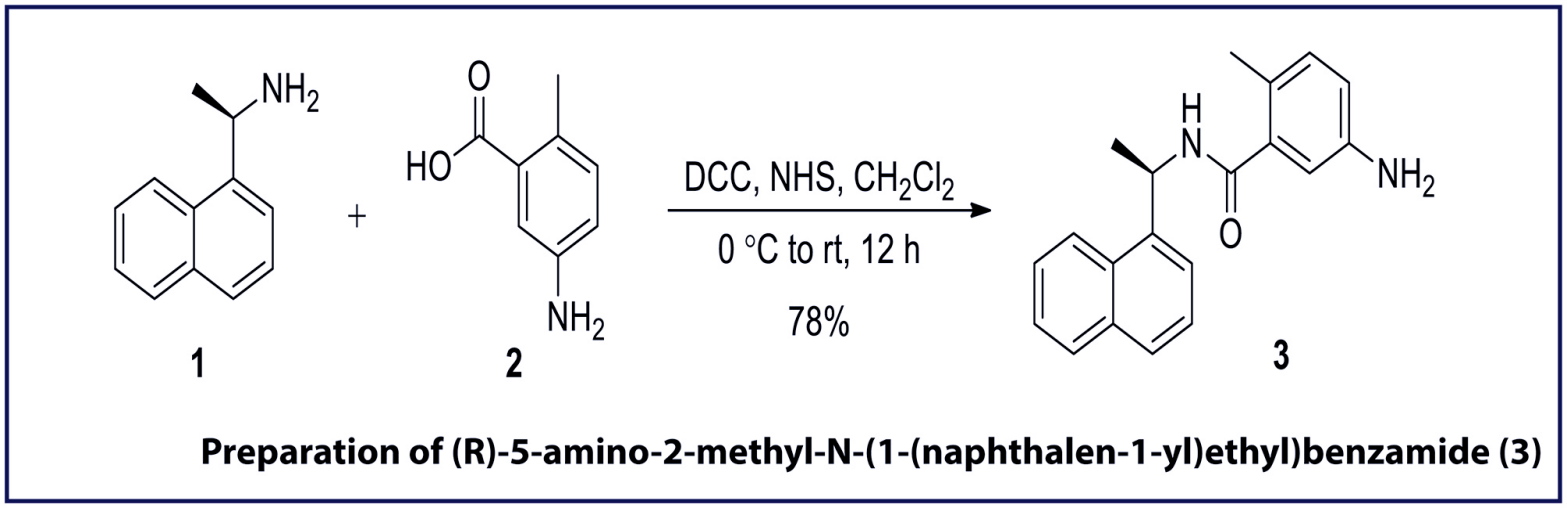
Chemical synthesis of GRL0617 inhibitor, a known inhibitor of SARS-CoV-2 PLpro activity. DCC, *N, N*-dicyclohexylcarbodiimide; NHS, *N*-hydroxysuccinimide; rt, room temperature. The figure is self-explanatory; the reagents and reaction conditions, i.e., experimental procedure is already mentioned on the arrow).

GRL0617 [(*R*)-5-amino-2-methyl-*N*-(1-(naphthalen-1-yl)ethyl) benzamide (Harcourt et al., 2004)] is an allosteric inhibitor of the PLpro and, as shown in Figure 2, the same was synthesised *in-house* for use as a reference control in enzyme-inhibition assays. For this preparation of **3**: To a cooled (0°C) and stirred solution of 5-amino-2-methylbenzoic acid **2** (25 mg, 0.16 mmol) in dry CH_2_Cl_2_ (10 mL), *N, N*-dicyclohexylcarbodiimide (31 μL, 0.20 mmol), and *N*-hydroxysuccinimide (22 mg, 0.20 mmol) were added in sequence. After stirring the reaction mixture for 10 min at the same temperature, (*R*)-(+)-1-(1-Naphthyl)ethylamine **1** (32 μL, 0.20 mmol) was added to the reaction mixture. The reaction mixture was stirred for 30 min at 0°C and then slowly warmed to room temperature. The progress of reaction was monitored by TLC and after the reaction was complete (12 h), the reaction mixture was filtered through the pad of celite. The celite pad was washed with CH_2_Cl_2_ (5 ml × 3), and the combined filtrate was washed sequentially with 1N HCl (15 ml), aqueous sodium bicarbonate solution (15 ml), and distilled water (15 ml). The combined organics was dried over Na_2_SO_4_, filtered, and evaporated to afford a crude residue. The crude residue obtained was purified by silica gel column chromatography (eluting with 40% ethyl acetate in hexane) furnished (*R*)-5-amino-2-methyl-*N*-(1-(naphthalen-1-yl)ethyl)benzamide 3 (39.26 mg, 78%) as a white solid. *R*_*f*_ = 0.29 in 50% ethyl acetate solution. ^1^H NMR and ^13^C-NMR chemical shifts has been reported per million (ppm) relative to the peak of residual proton signals of (*R*)-5-amino-2-methyl-N-(1-(naphthalen-1-yl)ethyl)benzamide 3 (400 MHz, CDCl_3_ and 100 MHz, CDCl_3_) shown in Supplementary Figures 5, 6.

### SCoV-2 PLpro Inhibition Assay

The test compounds and the reference control, GRL0671, were serially diluted starting from a maximum concentration of 10 mM and each dilution was mixed with 100 nM concentration of recombinant SCoV-2 PLpro in 96-well plates and incubated at 37°C for 45 min. After the 45 min incubation, reaction was initiated by adding the peptide-AMC substrate to a final concentration of 60 μM per well at 37°C for a further 45 min. Finally, the AMC release is measured by the fluorescence at 460 nm using the microplate reader. The 50% inhibitory concentration (IC_50_) of each drug was calculated by the dose-response inhibition function and curve-fitting using Graphpad Prism 8.0.1 with inhibitor vs normalised response equation. All inhibition assays were performed in duplicates (Sargsyan et al., 2020b; Fu et al., 2021; Osipiuk et al., 2021; Zhao et al., 2021).

### Antiviral Assays

#### Cell Cultures and Virus Strain

Human Lung Fibroblast (MRC-5, Cat # ATCC® CCL-171™) cell line was purchased from American Type Culture Collection (ATCC, USA). The MRC-5 cell line was maintained in Eagle's Minimum Essential Medium (EMEM) supplemented with 10% heat-inactivated Fetal Bovine Serum (FBS), 2 mM L-Glutamine and 0.1% Antibiotic-Antimycotic (ABAM) at 37°C in an incubator maintained to have 5% CO_2_. The assay medium consisted of EMEM supplemented with 2% FBS along with all other components of the growth medium. Human Coronavirus strain 229E (HCoV-229E, ATCC®VR-740™) was purchased from ATCC and propagated in MRC-5 cells. The stock virus was titrated by a plaque assay on MRC-5 cell monolayers using the standard protocol.

#### MTT Assay

Cytotoxicity and the antiviral activities of the selected drugs were estimated by MTT [(3-(4,5-dimethylthioazol-2-yl)-2,5-diphenyl tetrazolium bromide)]-based quantitative colorimetric assay in MRC-5 cell line in 96-well tissue culture plates. Briefly, cells were seeded at a density of 2 × 10^4^ cells/well in a 96-well tissue culture plate and grown to ≥80% confluency. At this stage, growth medium was replaced with 2-fold serial dilutions of the test compound prepared in the assay medium with each dilution added to six replicate wells at 50 μL/well of the 6 replicate wells, and three wells each were used for the estimation of cytotoxicity and the antiviral activity. Accordingly, wells meant for cytotoxicity estimation received 100 μl/well of assay medium, whereas those meant for estimation of antiviral activity received 100 μl/well of HCoV-229E (~500 PFU/well). Each assay plate included medium-only controls, cell controls and virus-infected cell controls, and the plates were incubated for 6 days, at which time point ≥90% cells in the virus control wells demonstrated CPE. Post incubation, culture supernatants from wells meant for the estimation of antiviral activity were collected for qPCR-based estimation of viral loads, while those from wells meant for the estimation of cytotoxicity were discarded, followed by replacing all the wells with 100 μl/well of MTT solution (0.5 mg/well) in assay medium. The plates were incubated for 4 h at 37°C to allow MTT-formazan formation and then supplemented with 100 μl/well formazan-solubilisation solution (10% SDS in.01 M HCl solution) and the plates were further incubated for 4 h at 37°C. The optical densities were recorded using a TECAN microplate reader at dual wavelengths of 570 and 690 nm, and the OD_570_-OD_690_ data values were calculated. For data analyses, the background-corrected optical densities in sample wells were obtained by subtracting the mean OD_570_-OD_690_ of medium controls in each plate from test samples (OD_570_-OD_690_ of Sample well - Mean OD_570_-OD_690_ of Medium control). The percent cytotoxicity and percent antiviral activity were calculated as follows:


$$\begin{array}{l} \%\text{ Cytotoxicity}=\frac{{\text{OD}}_{\text{57}0-\text{69}0}\text{ Sample Well }\times \text{ 1}00}{\text{Mean }{\text{OD}}_{\text{57}0-\text{69}0}\text{ Untreated Cell Control}}\\ \%\text{ Antiviral Activity}=\frac{\begin{array}{c} ({\text{OD}}_{\text{570}-\text{690}}\text{Sample Well}\\ -\text{Mean }{\text{OD}}_{\text{570}-\text{690}}\text{ Virus Control})\;\times \text{100} \end{array}}{\begin{array}{c} (\text{Mean }{\text{OD}}_{\text{570}-\text{690}}\text{ Untreated Cell Control}\\ -\text{Mean }{\text{OD}}_{\text{570}-\text{690}}\text{ Virus Control}) \end{array}} \end{array}$$

The data were plotted using Origin 6 (Microcal^TM^ Origin® version 6.0, Microcal Software Inc.) software and the 50% cytotoxicity concentration (CC_50_) and 50% effective concentration (EC_50_) for each compound at various concentrations were determined. Finally, the selectivity index (SI) for the test compound was determined from CC_50_/EC_50_ values.

#### Quantitative PCR

Cell-free culture supernatants were collected from virus-infected and drug-treated wells and were used to estimate the viral loads using SYBRGreen-based qRT-PCR targeting a 70-base region in the cDNA coding for the viral membrane protein. For this, 200 μl virus sample was processed to prepare the viral genomic RNA using HiPure™ Viral Nucleic Acid extraction kit (Cat # 11858874001; Roche Diagnostics, India). One-third of the viral genomic RNA preparation was reverse-transcribed as per manufacturer's instructions to synthesise the cDNA using ImProm-II Reverse Transcriptase (Cat # A3802, Promega Corporation, India). One-fourth of the cDNA preparation and the primer set, MBA134 (Forward) 5′-TTCCGACGTGCTCGAACTTT-3′ and MBA135 (Reverse) - 5′-CCAACACGGTTGTGA CAGTGA-3′, were used in quantitative PCR(qPCR) using TB Green® Premix Ex Taq™ II (TliRNaseH Plus) (Cat # RR820A, DSS-TAKARA) according to the manufacturer's recommendations. Recombinant plasmid DNA carrying the target sequence was generated for use in qPCRs as reference standard and its copy number was determined using URI Genomics and Sequencing Center's online tool. Serial 10-fold dilutions of the recombinant plasmid were used as templates in qPCRs and the Ct-values were used to generate the standard curve. A Ct value ≥ 30 was considered negative and the viral loads in test samples were derived from the standard curve. Viral loads in the test samples, represented as the viral genome equivalents (GE), were plotted against the drug concentrations tested in the assays. Similarly, viral loads in the test samples were compared with the virus controls to calculate percent reduction and Log_10_ reduction in the viral load.

### SARS-CoV2 Antiviral Testing

#### Cytotoxicity Assay

A test compound that demonstrated activities in PLpro protease-inhibition assays as well as in cell-based antiviral assays against HCoV-229E in MRC-5 cells was selected to demonstrate antiviral effects against SCoV-2 in Vero E6 cells *in vitro*. Because we had no physical access to a BSL-3 facility to carry out the antiviral assays against SCoV-2, this part of the work was outsourced to the Regional Center for Biotechnology (RCB), Faridabad, India. Initially, to determine the cytotoxicity of the selected compound, ~10^4^ Vero E6 cells were seeded per well of a 96-well plate and grown to ≥80% confluency. At this stage, cells were incubated for 30 h with dilutions of the test compound in replicate wells and these assays included uninfected cells, DMSO and Remdesivir (reference anti-SCoV-2 control)-treated wells as known negative and positive controls. Post-30 h incubation, cells were stained with the fluorescent (Hoechst 33342 and Sytox orange) dyes. The microscopic images were captured at 10 × magnification (16 images/well) using ImageXpress Micro Confocal (Molecular Devices).

#### Immunofluorescence Antiviral Screening Assay

The antiviral activity of the selected compound against SCoV-2 was analysed by immunofluorescent assay in a 96-well plate. For this, ~10^4^ Vero E6 cells per well were seeded and grown to ≥80% confluency. At this stage, cells in replicate wells were treated with a safe dose (identified from the cytotoxicity studies) of the selected compound or with 10μM Remdesivir. These assays also included the necessary negative controls for comparison. The cells were infected with SCoV-2 at 0.1 MOI and, post-30 h incubation, fixed in 4% paraformaldehyde, permeabilized with 0.3% Tween-20 and stained with anti-mouse mAb specific to SCoV-2 Nucleocapsid (primary) followed by staining with Alexafluor 568-labelled anti-mouse antibody (secondary). Prior to mounting, monolayers were treated with Hoechst 33342 to stain the nuclei and the images were captured using ImageXpress Micro Confocal (Molecular devices) at 10 × magnification (16 images/well). The data was analysed in MetaXpress software using a multi-wavelength cell scoring module and the cells double-positive for the viral nucleocapsid as well as nuclei were counted and compared with that in the controls.

### Statistical Analysis

All the experiments were performed in triplicates and the numerical data from these experiments was represented as Data Mean ± Standard Deviation (SD). The 50% cytotoxic concentrations (CC_50_) and 50% effective concentrations (EC_50_) of the compounds were calculated by modelling the data sets using logistic regression model, while the R-values and the statistical significance among data sets were derived from linear regression curves and the one-way ANOVA, respectively. A statistical significance of *p* < 0.05 was considered significant in all the experiments.

## Results

### Virtual Screening and Characterization of FDA-Approved Drugs Against SCoV-2 PLpro

#### Virtual Screening

A library of 2,467 FDA-approved compounds from the Drug bank database was virtually screened in HTVS mode against the modelled SCoV-2 PLpro. After following the criteria described in the *Materials and methods* section, a total of 12 potential compounds were selected for further characterization. These molecules were selected based on docking score, glide energy, and interactions with the active site residues of PLpro which were further subjected to MD simulations to assess the stability of the drug-receptor complex, and for the estimation of binding energy. The docking score, glide energy, and the interactions of the 12 selected molecules are provided in Table 1.

#### Molecular Dynamics (MD) Simulation Studies

The interactions of the 12 selected molecules with the active site residues of SCoV-2 PLpro were analysed under physiological conditions through MD simulation. The conformational changes associated with the protein-ligand interactions were depicted through Root-mean-square deviation (RMSD) plots as shown in Supplementary Figure 1. The backbone RMSD in the presence of different ligands showed negligible deviation from control indicating minimal influence of the bound molecules on the global structure of PLpro which is also evident from the radius of gyration plots (Supplementary Figure 2). The ligand RMSD variations are provided in Supplementary Table 1.

**Figure 3:**
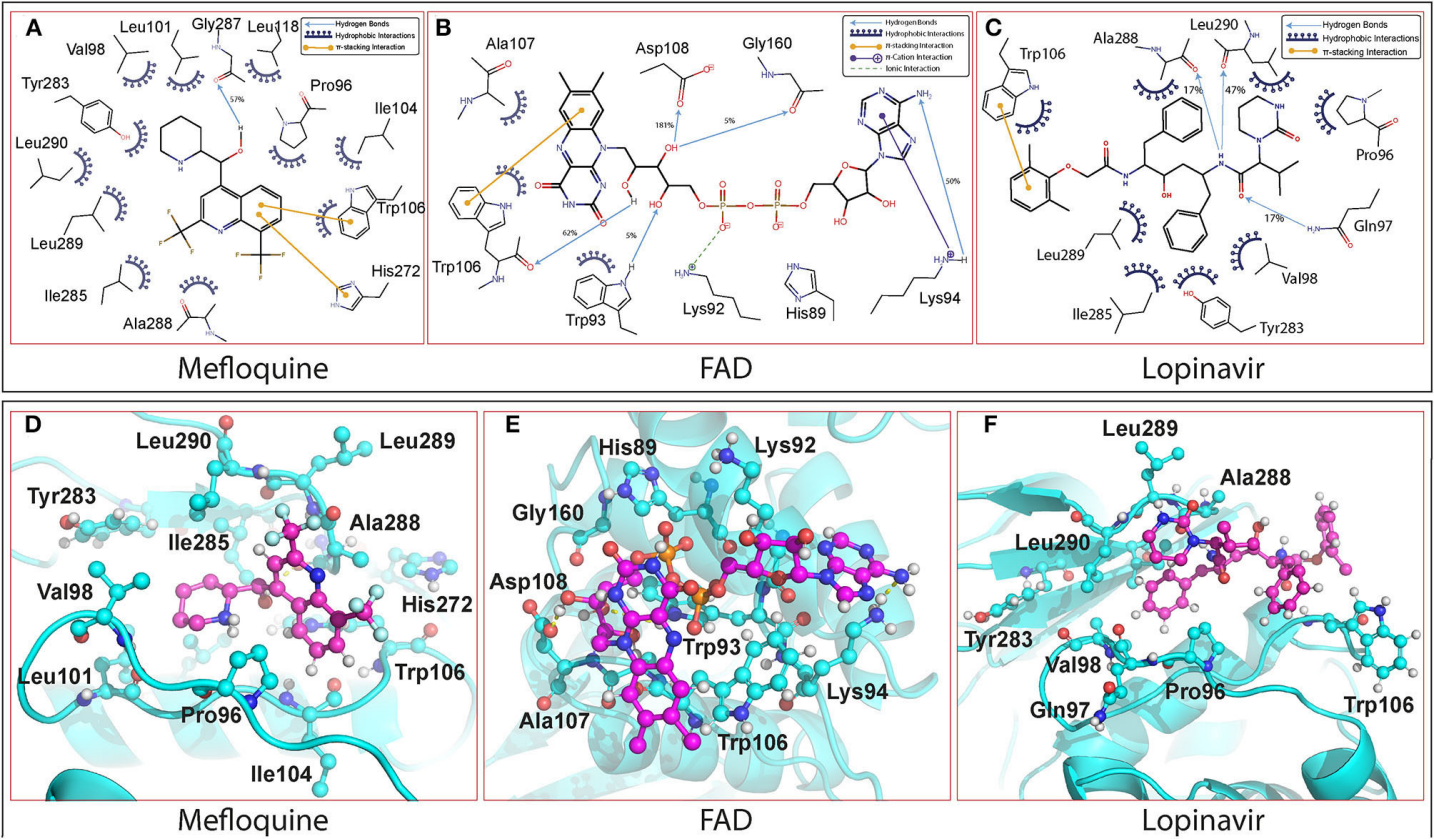
The interactions of the molecules with PLpro observed throughout the 100 ns simulations are depicted. The interactions were estimated by geometric criteria and visual inspection. (I) 2-D Interaction diagram of **(A)** Mefloquine, **(B)** FAD, and **(C)** Lopinavir with PLpro of SARS-CoV-2. (II) 3D-binding pose of **(D)** Mefloquine, **(E)** FAD, and **(F)** Lopinavir with PLpro of SARS-CoV-2.

The MD trajectory analysis of the 12 enzyme-drug complexes showed differential binding interactions of hits. Acarbose formed extensive hydrogen bonds with various active site residues including Trp106 and Asp286 owing to the high number of hydrogen bond donors and acceptors. But the molecule was significantly solvent-exposed due to its high polarity and absence of hydrophobic interactions. Similarly, in the case of Flavin adenine dinucleotide (FAD), hydrogen bonds were observed with residues like Trp106 and Asp286 among others. Also, the flavin group and the adenyl ring allowed the formation of π-stacking interactions with Trp106 and other hydrophobic interactions with Trp93 and Ala107 as shown in Figures 3B,E. Although the phosphate group formed ionic interaction with Lys92, the hydrophilic sugar and phosphate of FAD were predominantly solvent-exposed during the simulation. Glutathione disulphide was bound to the protein by forming extensive hydrogen bonds with various residues including Asp108 and His272 and these interactions were retained throughout the simulation. The molecule was anchored to the binding site by virtue of hydrophobic interactions between the disulfide of glutathione and residues Trp106 and His272. Although this molecule resided in the active site during the simulation, it fluctuated due to the high number of rotatable bonds.

Among the 12 selected hits, Mangafodipir displayed the best interaction with SCoV-2 PLpro with greater penetration into the active site. Besides forming hydrogen bonds with Asp286 and Cys111, it also formed π-stacking interactions with His272 and Trp106 in addition to van der Waal's contacts with several non-polar residues in the binding site and retained them throughout the 100 ns simulation. Rutin was decently stable in the binding site as the flavenone group formed π-stacking interactions with Trp106 and His272 while the polar sugar moiety formed hydrogen bonds with Asp286 and others as shown in the interaction table. The binding of Ritonavir was stable in the active site through π-stacking interactions between its thiazole ring and residues Trp106 and His272. Its interactions with the enzyme were further stabilised through hydrophilic interactions with residues Asp108 and Asn109 along with hydrophobic interactions with Ala107, Tyr112, and other residues. Steviolbioside showed intermediate interactions with the protein-forming hydrogen bonds with residues Asp286 and Trp106. The steviol group maintained hydrophobic interactions with residues Leu289, Pro96, Ala107, and Ala288. The binding of Darunavir with PLpro was unsteady due to high solvent exposure. It formed meagerly stable interactions which included hydrogen bonds with Gln289, Cys270, and hydrophobic interactions with Leu162.

Lopinavir and Mefloquine occupied the hydrophobic pocket formed between β11 loop and α7 loop. While Lopinavir showed a relatively variable binding position, Mefloquine was quite stable in the pocket. Along with hydrogen bonds, both molecules majorly formed hydrophobic interactions with binding pocket residues including stacking interactions with Trp106. Detailed interactions of Lopinavir and Mefloquine are depicted in Figure 3. The molecules Iopromide and Lactitol were highly unstable in the binding site in the absence of specific interactions with the protein. Although Iopromoide was anchored by hydrogen bond with Asp108, Lactitol got completely dissociated from the active site after initial 30 ns of simulation. The interacting residues observed throughout the 100 ns simulations for all the molecules are listed in Supplementary Table 2.

#### Binding Free Energy Analysis by MM-PBSA Method

The binding free energy values were computed from the last 25 ns trajectory using the MM-PBSA approach. The binding energy values were in coherence with the interactions observed and therefore gave a plausible binding affinity of the hits. The lowest binding free energy values were observed for Mangafodipir and Glutathione followed by Ritonavir, FAD, Lopinavir, and Mefloquine. These molecules also displayed significant hydrogen bonds and hydrophobic interactions and the majority of them were retained throughout the 100 ns simulations. The molecules Acarbose, Iopromide, Steviolbioside, and Darunavir were more solvent-exposed and therefore exhibited higher binding free energies. Although some of them displayed decent hydrogen bonding, the higher binding free energy values were due to the lack of effective hydrophobic interactions. Binding free energy could not be computed for Lactitol and binding energy of all the molecules listed in Supplementary Table 3.

#### HCoV-229E and SCoV-2 PLpro Are Structurally Similar

Due to lack of physical access to a functional BSL-3 facility, we wanted to use HCoV-229E as a model coronavirus to investigate the antiviral potential of the selected molecules in a cell-based assay system. Accordingly, HCoV-229E PLpro was modelled to study the structural similarity with the SCoV-2PLpro. Although the HCoV-229E PLpro shares lesser sequence identity (~22%) with SCoV-1 and SCoV-2 PLpro at the nucleotide level, it exhibits ~53% similarity with SCoV-2 PLpro at the amino acid level. The 3D structure of HCoV-229E PLpro was modelled using I-TASSER (threading-based structure prediction). Validation of the models was done based on stereochemical parameters of the model (MolProbity). Comparison of the SCoV-2 PLpro and HCoV-229E PLpro shows that the two structures are significantly similar with RMSD of ~1.7 Å, but deviated in the C-terminal tail region (Supplementary Figure 4A). Also, most of the catalytic residues are conserved or similar in both the structures except tryptophan to threonine at residue 106 (Supplementary Figure 4B). The 12 identified molecules were also docked into HCoV-229E PLpro and they showed similar binding conformation as observed with SCoV-2 PLpro, and their docking score, glide energy and molecular interactions are shown in Supplementary Table 4.

#### Signalling Pathways/Molecular Mechanisms Between HCoV-229E and SCoV-2 Are Similar

The sequence analysis of the two genomes suggested 55.3% identity between the two with a GC content of 38%. Except for some short genomic regions, the majority of the genomic regions demonstrated pairwise identity between 30 and 100% (van der Hoek et al., 2004; Woo et al., 2005). Although SCoV-2 and HCoV-229E enter into host cells *via* different receptors, ACE2 and APN respectively, they both require type II transmembrane for the activation of Spike protein. Moreover, the available literature distinctly demonstrates the similarities in various pathways between the two viruses including the unfolded protein response, the MAPK pathway and the proinflammatory response pathways (Tyrrell, 1965; Weiss, 2005). From the literature survey, we found that the signalling pathways involved in disease pathogenesis and host immune response are mostly similar (Siddell et al., 1983; Masters, 2006; Weiss and Leibowitz, 2011). The host immune response proteins IFITMs (interferon inducible transmembrane proteins) implicated in the attachment and entry of SCoV-2 and HCoV-229E are conserved. Also, SCoV-2 and HCoV-229E induce ER stress/unfolded protein response, MAPK signalling pathways and the activation of innate immune responses through similar molecular mechanisms (Fung and Liu, 2019). Interferons (IFNs) secreted by the infected cells play a major role in antiviral innate immune defence mechanisms through the expression of IFN-stimulated genes (ISGs) which restrict viral replication in a well-coordinated manner. Accordingly, Pfaender et al. found that ISGs inhibited the infection of both SCoV-2 and HCoV-229E, though the efficiencies varied (Pfaender et al., 2020). It was observed that several ISGs, like APOL2, RAB27A, FAM46A, and FAM46C that inhibited SCoV-2 replication, also inhibited HCoV-229E replication. However, other ISGs like SQLE, SLC1A1, and STARD5, which were found to potentially inhibit HCoV-229E, were not found to inhibit SCoV-2 (Zhao et al., 2022). These studies demonstrated overlap in ISG-mediated inhibition mechanisms of SCoV-2 and HCoV-229E replication. Overall, the available literature provides enough evidence suggesting the existence of similarities in viral and host pathways involved in HCoV-229E and SCoV-2 replication cycles, and therefore support the use of HCoV-229E as a suitable surrogate in drug-discovery studies (Ma et al., 2020; Butot et al., 2021; Owen et al., 2021; Pasquereau et al., 2021).

### Bacterially Expressed SCoV-2 PLpro Was Proteolytically Active *in vitro*

**Figure 4:**
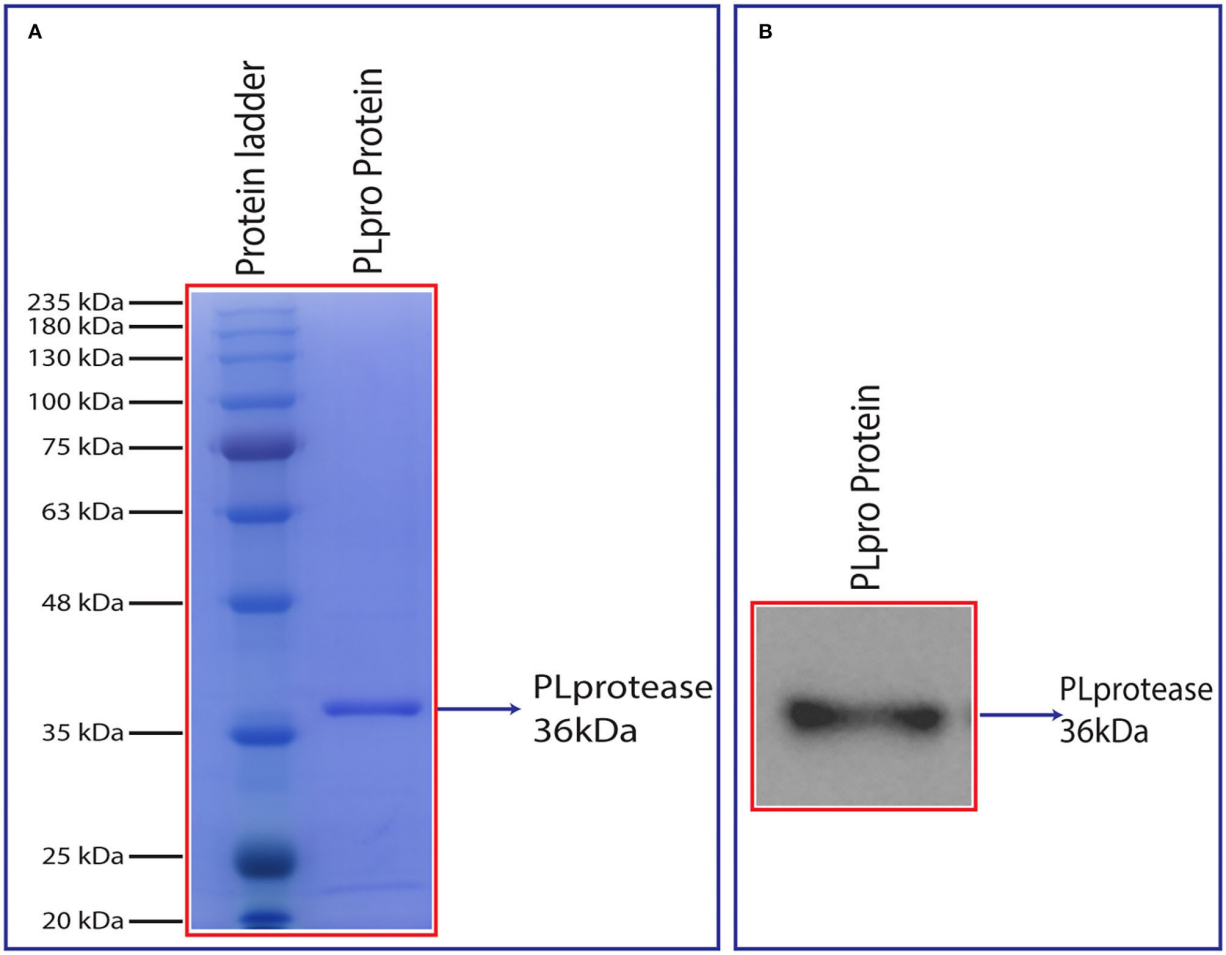
**(A)** SDS-PAGE analysis of purified PLpro after size exclusion chromatography and **(B)** chemiluminescent detection of the recombinant PLpro using anti-His IgG (6x) antibody in a western blot. Synthesized PLpro clones were transformed, expressed in 2X YT media at 18°C with 0.5 mM IPTG and 1 mM ZnCl_2_ for 20 h. The harvested pellet was lysed, sonicated, and centrifuged at 19,496 g for 1 h. The cell supernatant was passed through HisTrap FF column and protein eluted with linear gradient imidazole. The eluted fractions were analysed by SDS and western blot followed by the protein that was loaded into HiLoad 26/600 Superdex 200 pg column for further purification. The purity of the protein was analysed using 12% SDS-PAGE and confirmed by western blot (~36 kDa).

**Figure 5:**
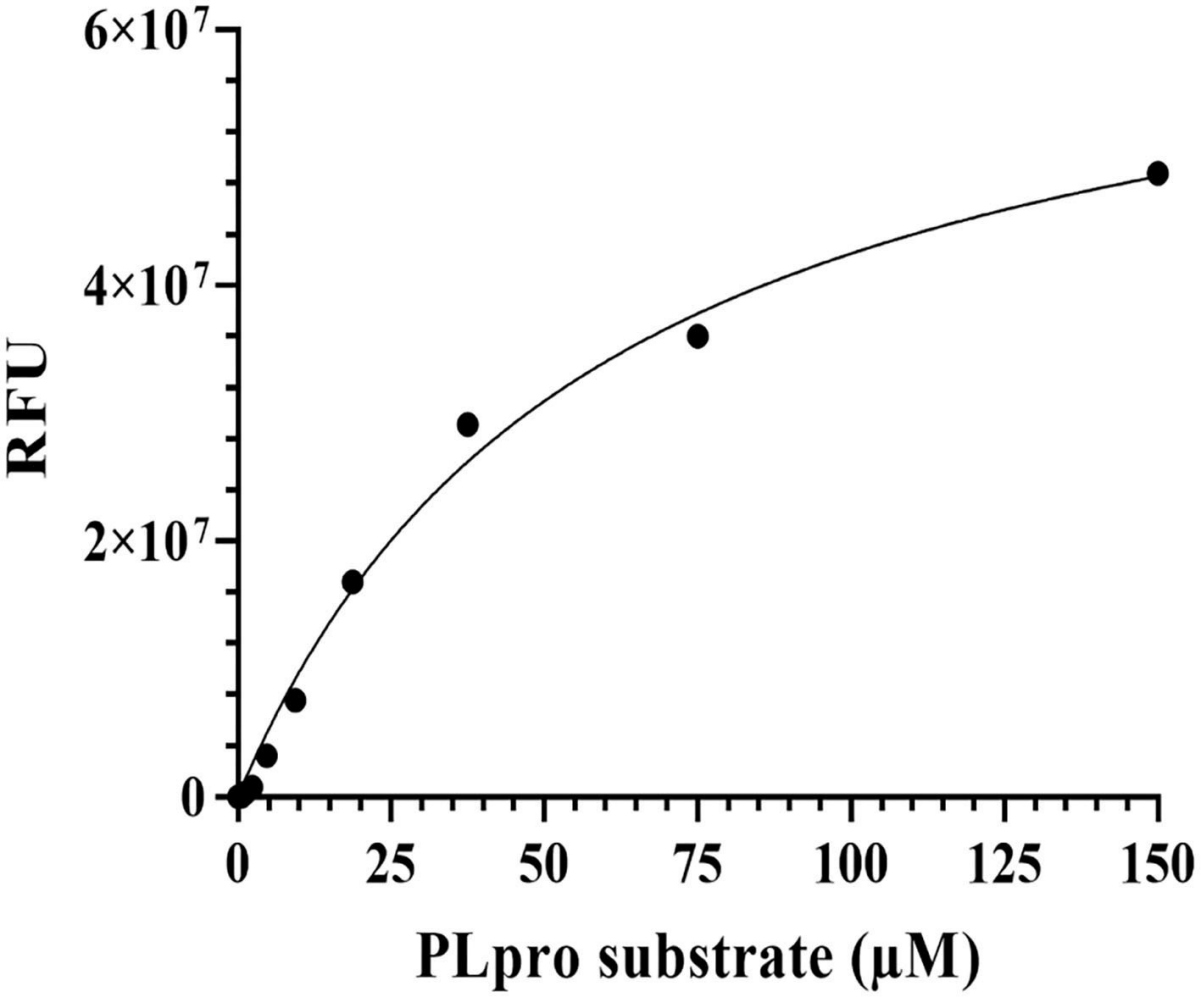
Optimization of PLpro activity assay using Michaelis-Menten equation. PLpro enzymatic activity assay was performed using the fluorescence substrate peptide-AMC in 50 μL reaction volume in 20 mM Tris pH 7.5, 100 mM NaCl, and 2 mM DTT buffer conditions at 25°C. The PLpro protein reaction was kept with increasing concentrations of PLpro substrate and incubated at 37°C for 45 min in biological replicates. After incubation, the fluorescence signals were monitored at 460 nm (excitation at 360 nm) and graphs were plotted in GraphPad prism software.

The cDNA coding for SCoV-2 PLpro (~1,286 bp) in pET22b was codon-optimised and custom-synthesised at GeneScript, USA. The bacterially expressed and purified PLpro protein had a molecular weight of ~36 kDa on 12% SDS-PAGE and the same was confirmed by western blot assay using anti-His antibody (Figure 4). The enzymatic activity of purified SCoV-2 PLpro was assayed as described (Materials and Methods section). The recombinant enzyme demonstrated significant activity at 100 nM and therefore, this concentration was used for further experiments. To determine the optimum amount of the substrate for enzyme assay, we carried out substrate optimization by varying its concentration (0-150 μM) in the reaction with 100 nM PLpro and the calculated K_m_ of the peptide-AMC substrate was found to be 60 μM (Figure 5). Accordingly, a substrate concentration of 60 μM of the peptide-AMC and a concentration of 100 nM of the SCoV-2 PLpro were chosen for use in protease-inhibition assays to estimate the IC_50_ of the selected FDA-approved compounds.

### FAD Hydrate and Mefloquine Effectively Inhibited SCoV-2 PLpro Activity

**Figure 6:**
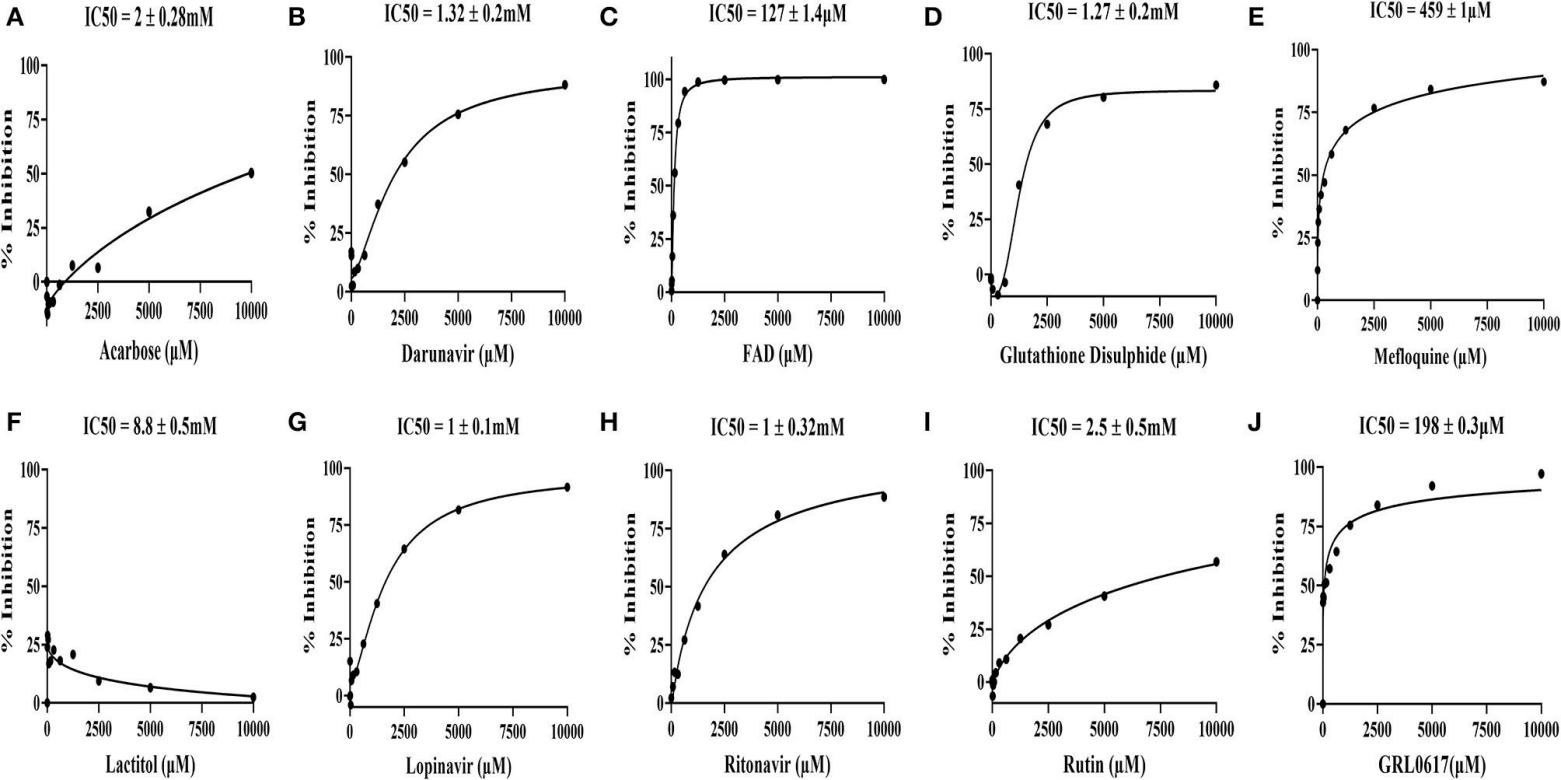
*In vitro* inhibitory activity of the drugs identified through the virtual screen of the FDA-approved drug library along with GRL0617 inhibitor, a known antagonist of SARS-CoV-2 PLpro. **(A)** Acarbose, **(B)** Darunavir, **(C)** FAD hydrate, **(D)** Oxidized L-Glutathione disulphide, **(E)** Mefloquine-HCl, **(F)** Lactitol, **(G)** Lopinavir, **(H)** Ritonavir, **(I)** RutinTrihydrate, and **(J)** GRL0617 (5-amino-2-methyl-N-[(1R)-1-naphthalen-1-ylethyl]benzamide). The identified nine molecules with GRL0617 molecules were diluted (10-0 mM) and incubated with PLpro at 37°C for 45 min. After incubation, peptide-AMC substrate reaction was added at 37°C for 45 min to initiate the reaction. Finally, release of the AMC was measured by excitation λ: 364 nm; emission λ: 440 nm using a microplate reader. The IC_50_ values were calculated by the dose-response-inhibition function by GraphPad prism software. The assay experiments were performed in biological duplicatives.

Of the 12 molecules selected from the FDA-approved drug library, Iopromide, Steviolbioside, and Mangafodipir were not considered for further testing based on the computational output and logistic issues. The GRL0617, an established SCoV-2 PLpro inhibitor, was synthesised *in-house* (Figure 6J). ^1^H NMR of (*R*)-5-amino-2-methyl-N-(1-(naphthalen-1-yl)ethyl)benzamide 3 (400 MHz, CDCl3): δ 8.17 (d, J = 8.4 Hz, 1H), 7.87 (d, J = 7.6 Hz, 1H), 7.82 (d, J = 8.0 Hz, 1H), 7.63 (d, J = 8.0 Hz, 2H), 7.59 (d, J = 7.2 Hz, 1H), 7.54–7.45 (m, 3H), 7.17 (d, J= 8.0 Hz, 2H), 6.39 (d, *J* = 7.2 Hz, 1H), 6.12 (quintet, *J* = 6.8 Hz, 1H), 2.36 (s, 3H), 1.77 (d, *J* = 6.8 Hz, 3H) (Supplementary Figure 5). ^13^C NMR of (*R*)-5-amino-2-methyl-N-(1-(naphthalen- 1-yl)ethyl)benzamide 3 (100 MHz, CDCl_3_): δ 166.3, 141.8, 138.3, 133.9, 131.6, 131.2, 129.1, 128.7, 128.4, 126.9, 126.8, 126.6, 125.9, 125.2, 123.5, 122.6, 45.1, 21.4, 20.7 (Supplementary Figure 6). The ^1^H-^1^H COSY spectra (400 MHz, CDCl_3_)of (*R*)-5-amino-2-methyl-N-(1-(naphthalen−1-yl)ethyl) -benzamide 3 are shown in Supplementary Figure 7.

The SCoV-2 PLpro inhibition assays were performed using 100 nM of the purified recombinant SCoV-2 PLpro and 60 μM of the peptide-AMC in the presence of 0-10 mM of the nine selected FDA-approved drugs, which were commercially procured, and screened in 12-point titration experiments. All the selected drugs and the reference molecule inhibited SCoV-2 PLpro activity in a dose-dependent manner. As shown in Figure 6, the reference drug, GRL0617, effectively inhibited SCoV-2 PLpro activity with an IC_50_ value of 198 μM. Among the 9 test compounds, FAD hydrate demonstrated higher inhibitory activity on SCoV-2 PLpro with an IC_50_ value of 127 ± 1.4 μM (Figure 6C) followed by Mefloquine with an IC_50_ value of 459 ± 1μM (Figure 6E). All other drugs demonstrated moderate to poor inhibitory activities in the millimolar range with Lactitol (IC_50_ = 8.8 ± 0.5 mM) being the least effective, which failed to inhibit the protease activity even at higher concentrations and the same is in agreement with our *in silico* data. Among those that demonstrated moderate inhibitory activities were in the order of Lopinavir (IC_50_ = 1 ± 0.1 mM) > Ritonavir (IC_50_ = 1 ± 0.32 mM) > Glutathione disulphide (IC_50_ = 1.27 ± 0.2 mM) >Darunavir (IC_50_ = 1.32 ± 0.2 mM), while Rutin trihydrate (IC_50_ = 2.5 ± 0.5 mM) were poor inhibitors of SCoV-2 PLpro (Figure 6).

### Antiviral Activities Against a Model Human Coronavirus

All the 9 compounds that were assayed in the protease-inhibition assay were tested in cell-based assays for antiviral potential against the human coronavirus, HCoV-229E. Similarly, along with GRL0617, Favipiravir, a known antiviral drug having target specificity to the viral RdRp and that has been shown to be clinically effective in mild-to-moderate COVID-19 patients, was included as a known reference in these antiviral assays (Wang et al., 2020; Driouich et al., 2021; Manabe et al., 2021).

#### Cytotoxicity of the Selected Compounds

**Figure 7:**
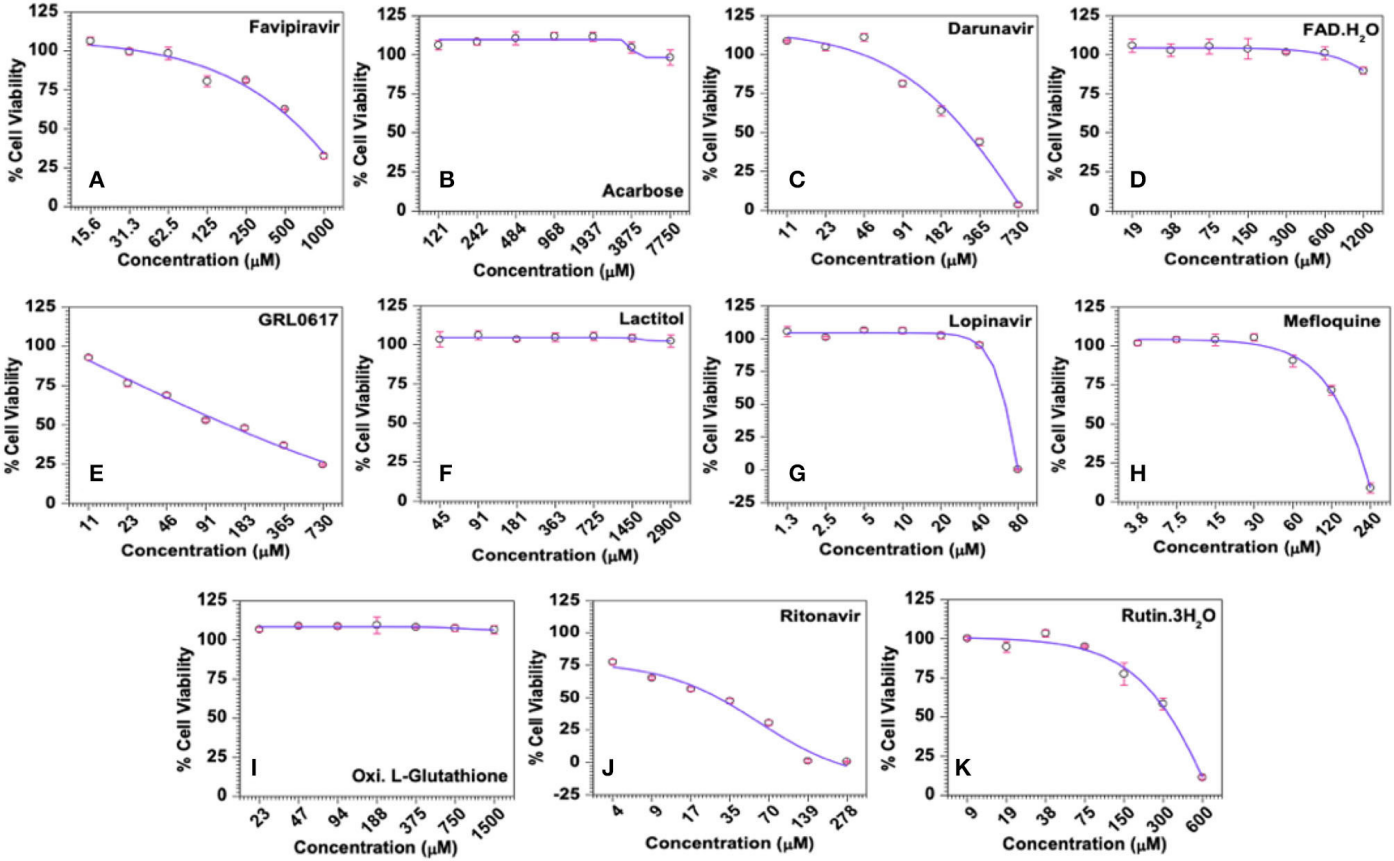
Cytotoxicity profiles of the selected compounds in MRC-5 cells *in vitro*. **(A)** Favipiravir (used as known reference control), **(B)** Acarbose, **(C)** Darunavir, **(D)** FAD hydrate, **(E)** GRL0617, **(F)** Lactitol, **(G)** Lopinavir, **(H)** Mefloquine, **(I)** Oxidized L-Glutathione, **(J)** Ritonavir, and **(K)** Rutintrihydrate were incubated with MRC-5 cells at the indicated concentrations for 6 days at 37°C in a CO_2_ incubator and the cell viability was analysed quantitatively by the colorimetric MTT assay as described in Methods. The raw data were converted to % cell viability and plotted against the indicated doses. Experimental data sets were modelled using a logistic regression model and the 50% cytotoxic concentrations (CC_50_) were derived.

Cytotoxicity, represented as 50% cytotoxic concentration (CC_50_), of the selected drugs was analysed in quantitative colorimetric MTT assays. As shown in Figure 7A, Favipiravir exerted dose-dependent cytotoxicity in MRC-5 cells and its calculated CC_50_ was 665 μM. Negative and higher *R*-value (*R* = −0.9375) along with higher score of statistical significance (*p* = 0.0018) supported statistical fitness of the regression model used for analysis, and the existence of statistically significant dose-dependent cytotoxicity. With regard to the test compounds, some of the compounds like, Acarbose, FAD hydrate, Lactitol, and Oxidized L-Glutathione, were largely non-toxic (maximum cytotoxicity <15%) across the tested concentrations, while others like Darunavir, GRL0617, Lopinavir, Mefloquine, Ritonavir, and Rutintrihydrate demonstrated distinct dose-dependent cytotoxicity in MRC-5 cells (Figures 7B–K). Though dose-dependent cytotoxicity by Acarbose, FAD hydrate, Lactitol, and Oxidized L-Glutathione was not obvious to the naked eye, negative *R*-values obtained for these compounds in the regression analyses suggested existence of an inverse relation between the dose of the compound and its cytotoxicity, thereby the existence of dose-dependent cytotoxic effects (Supplementary Table 5). Lower *R*-values and statistically weaker significance scores for these compounds, though reflected weaker relation, were expected as they exerted very little cytotoxicity, which fluctuated greatly within the smaller window of toxicity.

#### Mefloquine and Lopinavir Demonstrated Significant Antiviral Activity

The antiviral activity, represented as 50% effective concentration (EC_50_), is defined as the concentration of a drug that resulted in 50% protection of virus-infected cells from virus-induced cytopathic effects (CPE) and the same were derived from the quantitative colorimetric MTT assays performed in HCoV-229E-infected MRC-5 cells in the presence of reference controls or the test compounds. Overall, all the test compounds and the reference controls exerted dose-dependent antiviral activities and the positive *R*-values derived from the regression analysis suggested a direct relation between the dose of the drug and the antiviral activities. In these assays, Favipiravir was able to inhibit virus-induced CPE in about 17% of cells at a relatively safe concentration of 500 μM, but failed to show any obvious protection when treated with 2-fold lesser (250 μM) concentration suggesting a narrow range of antiviral activity, at least against HCoV-229E. One-way ANOVA analysis of the data suggested that the antiviral effects induced by the drug were statistically significant (*R* = 0.7933; *p* = 0.0333).

**Figure 8:**
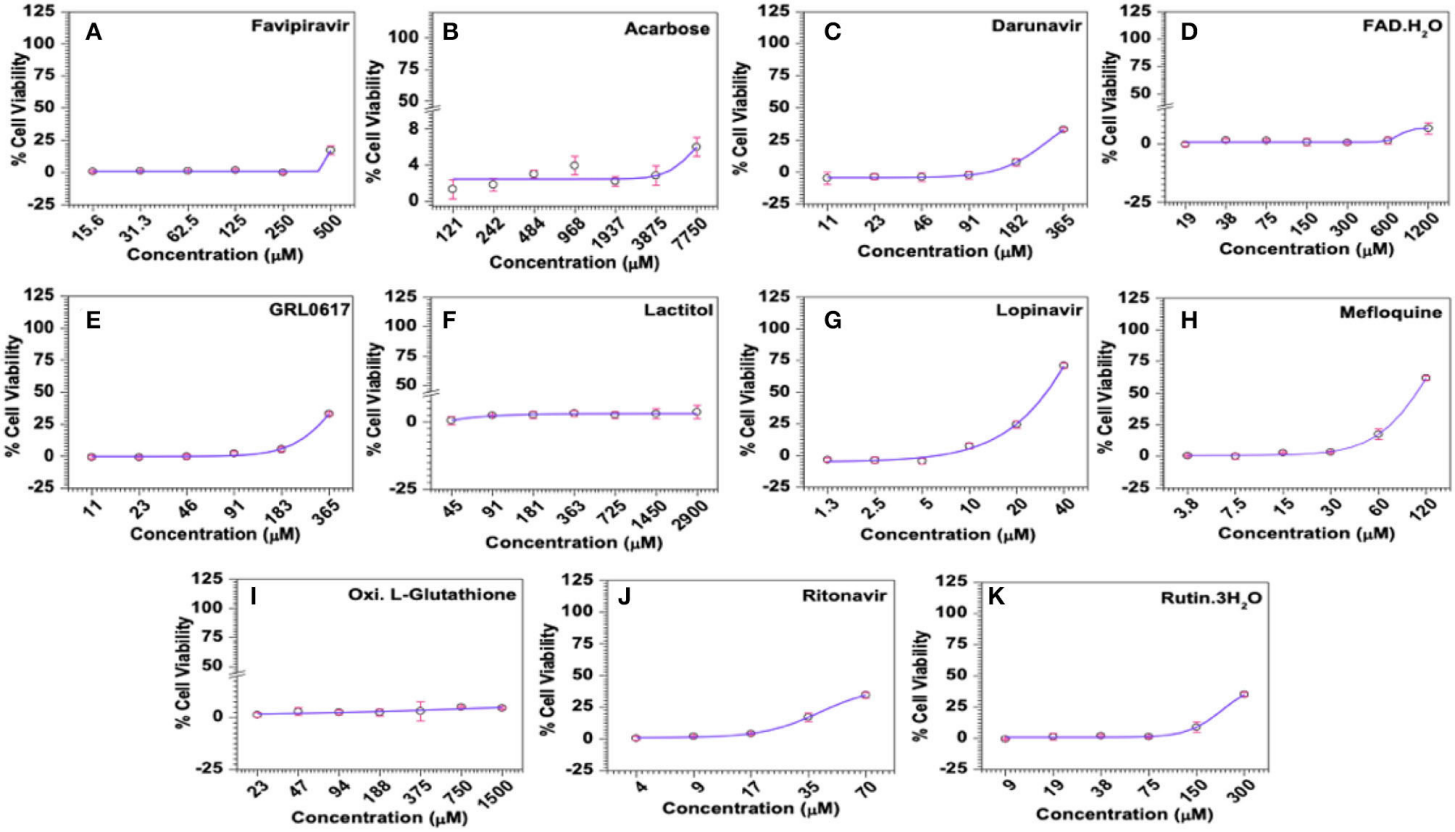
Antiviral activities of selected compounds in MRC-5 cells *in vitro*. **(A)** Favipiravir (used as known reference control), **(B)** Acarbose, **(C)** Darunavir, **(D)** FAD hydrate, **(E)** GRL0617, **(F)** Lactitol, **(G)** Lopinavir, **(H)** Mefloquine, **(I)** Oxidized L-Glutathione, **(J)** Ritonavir, and **(K)** Rutintrihydrate were incubated with MRC-5 cells at the indicated concentrations for 6 days at 37°C in a CO_2_ incubator and the antiviral activities of the compounds represented as the percentage of cells protected from virus-induced CPE was analysed quantitatively by the colorimetric MTT assay as described in Methods. The raw data was converted to % cell viability and plotted against the indicated doses. Experimental data sets were modeled using a logistic regression model and the 50% effective concentrations (EC_50_) were derived (Supplementary Table 4).

Similar to Favipiravir, Acarbose, FAD hydrate, Lactitol, and Oxidized L-Glutathione offered very little or no protection from virus-induced CPE within the safe concentrations tested in the study (Supplementary Table 5). Accordingly, the maximum antiviral effect exerted by these compounds was only about 8%. In contrast, Darunavir, Lopinavir, Mefloquine, Ritonavir, and Rutin trihydrate demonstrated significant dose-dependent antiviral activities against HCoV-229E in MRC-5 cells (Figure 8 and Supplementary Table 5). However, it should be noted that Darunavir and Ritonavir exerted their maximum antiviral effects of 33.6 and 34.5%, where the average cell viability was only 44.5 and 30.5%, respectively. Among others, treatment with Lopinavir (maximum ~71% at 40 μM) and Mefloquine (maximum ~62% at 120 μM) resulted in higher protection from virus-induced effects (Figures 8F,H and Supplementary Table 5). Calculated SI values for Lopinavir and Mefloquine were 2.03 and 1.6, respectively. Our data suggested that Lopinavir, with a higher SI value, is relatively safe and more potent compared to Mefloquine against HCoV-229E.

#### Mefloquine and Lopinavir Treatment Lead to Reduction in Viral Loads

**Figure 9:**
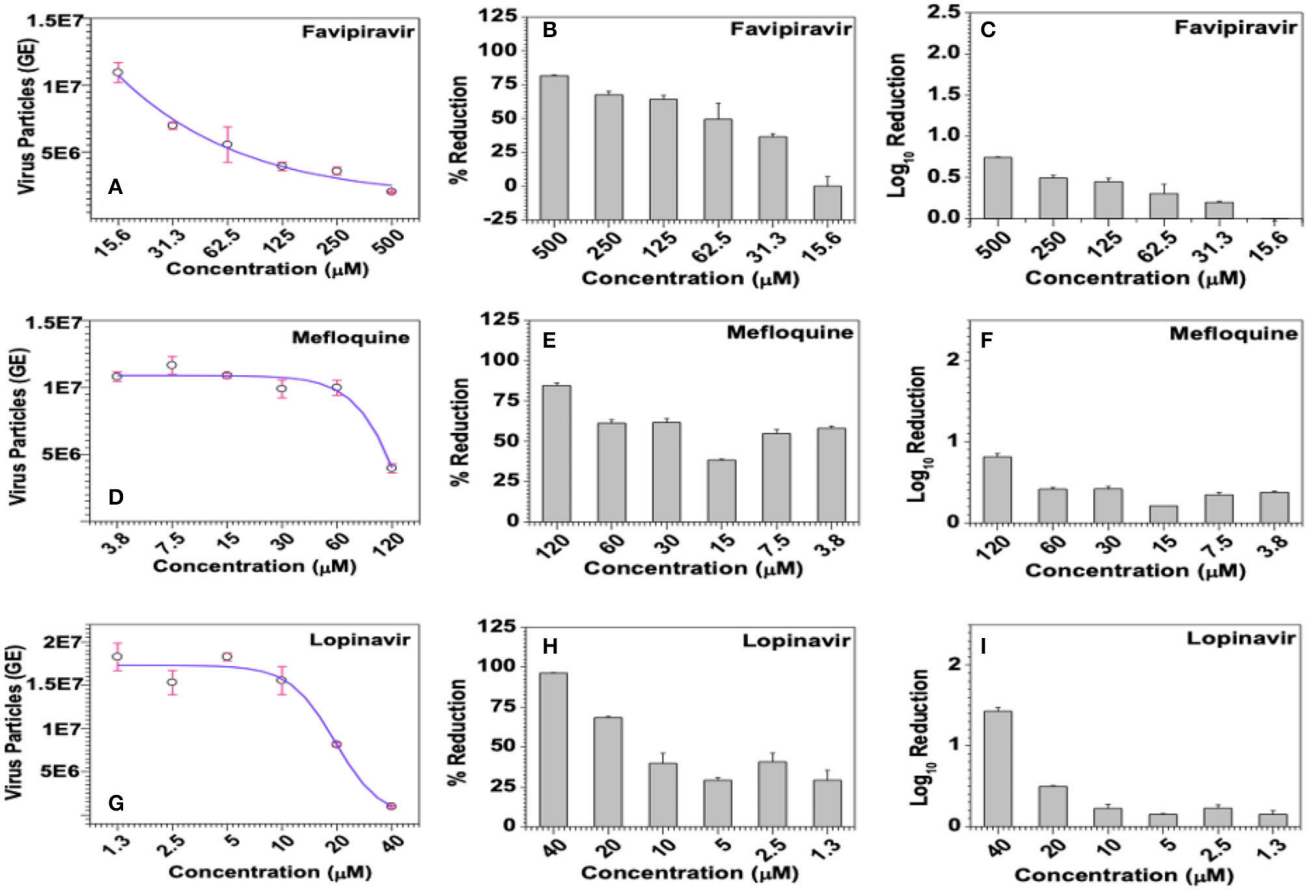
Effect of the selected compounds on virus titers. **(A–C)** Favipiravir, **(D–F)** Mefloquine, and **(G–I)** Lopinavir were incubated with HCoV-229E-infected (^@^1.0 MOI) MRC-5 cells at the indicated concentrations for 6 days, virus-infected culture supernatants from triplicate wells were individually processed to extract viral genomic RNAs and quantified by SYBR Green-based qPCR using primer pair targeting a 70-base region in the cDNA coding for the viral membrane protein. Ten-fold serial dilutions of the recombinant plasmid carrying the target 70-base region were used to generate the standard curve, titers of virus particles **(A,D,G)**, represented as genome equivalents (GE), were derived from the standard curve as detailed in methods and the data was used to calculate % inhibition **(B,E,H)**, and log_10_ inhibition **(C,F,I)** in viral titers.

Viral loads in infected culture supernatant samples were measured by SYBR Green-based quantitative PCR using the primer pair targeting a 70-base region within the cDNA coding for the viral membrane protein. Ten-fold serial dilutions of a recombinant plasmid carrying this 70-base target region was used to generate the standard curve and the same was used to derive the number of virus particles, represented as genome equivalents (GE), in the assay samples. In this study, Favipiravir exhibited dose-dependent downregulation of HCoV229E titers, as can be inferred from the negative *R* value (*R* = −0.9257) in the regression analysis, and reduced the viral loads by 0.75 log_10_ at a dose of 0.5 mM (Figure 9A). The observed dose-dependent reduction in viral titers due to Favipiravir treatment was also statistically highly significant (*p* = 0.0028) (Figures 9B,C).

Among the test compounds, Darunavir, Lopinavir, Mefloquine, Ritonavir, and Rutintrihydrate have demonstrated significant reduction in viral titers in a dose-dependent manner and the same is also evident from the negative *R*-values obtained in the regression analyses (Figures 9D,G and Supplementary Table 5). Accordingly, treatment with 40 μM Lopinavir or 365 μM Darunavir each resulted in about 96% reduction in viral titers, which translated into 1.42 log_10_ inhibition (Figures 9H,I and Supplementary Table 5). However, attributable to some fluctuations in estimated viral counts, dose dependency of Darunavir on HCoV-229E titers was only marginally significant (*R* = −0.8192, *p* = 0.0461), whereas the dose-dependent effects exerted by Lopinavir were statistically more significant (*R* = −0.8629, *p* = 0.0269). Similarly, treatment with 120 μM Mefloquine or 70 μM Ritonavir or 300 μM Rutin trihydrate lead to 84.7, 73.3, and 38.44% reduction in viral titers, which roughly translated into 0.8, 0.6, and 0.2 log_10_ inhibition, respectively (Figures 9E,F and Supplementary Table 5). However, statistically, dose dependent effects of Mefloquine were not significant (*R* = −0.7659, *p* = 0.0758), whereas those of Ritonavir (*R* = −0.9476, *p* = 0.0012) and Rutin trihydrate (*R* = −0.9452, *p* = 0.0044) were highly significant. With regard to GRL0617, a known inhibitor of coronavirus PLpro protease and a reference control in our PLpro enzyme assays, though demonstrated notable dose-dependent antiviral effects in the form of protection from virus-induced CPE in the MTT-based colorimetric assay (Figure 8E), the same was not found to have translated into dose-dependent reduction in viral titers (Supplementary Table 5). Accordingly, regression analysis too yielded a poor *R*-value (*R* = −0.0712), suggesting a larger variation in viral loads between samples treated with different doses of the drug. Further supporting lack of dose-dependent antiviral responses, one-way ANOVA of experimental data sets yielded highly insignificant (*p* = 0.8845) relation between the viral titers and the dose of GRL0617. Similar data was consistently obtained for GRL0617 against HCoV-229E in repeat experiments.

### Mefloquine Potentially Inhibits SCoV-2 Viral Replication in Live Cells

Among the nine selected FDA-approved inhibitors and those having SCoV-2 PLpro as their target, Mefloquine has shown promise as a potential drug both in PLpro-inhibition assays as well as against HCoV-229E in cell-based antiviral assays, which makes it a drug of choice for testing against SCoV-2.The optimised protocol to test the cytotoxicity and antiviral activity of a potential compound involves the use of Hoechst 33342, a blue fluorescent dye used to stain the nuclei of living or fixed cells, and the Sytox orange, an orange stain used to stain nucleic acids in cells with compromised membranes, an indicator of cell death. Further, Remdesivir at 10 μM was used as a reference control, at which concentration it is >99% safe in Vero E6 cells and also protects >99% cells from SCoV-2-induced CPE. In these experiments, Mefloquine demonstrated about 45% cytotoxicity at 5 μM concentration, but was largely safe (7% cytotoxicity) at a 10-fold diluted concentration of 0.5 μM (Supplementary Table 6).

**Figure 10:**
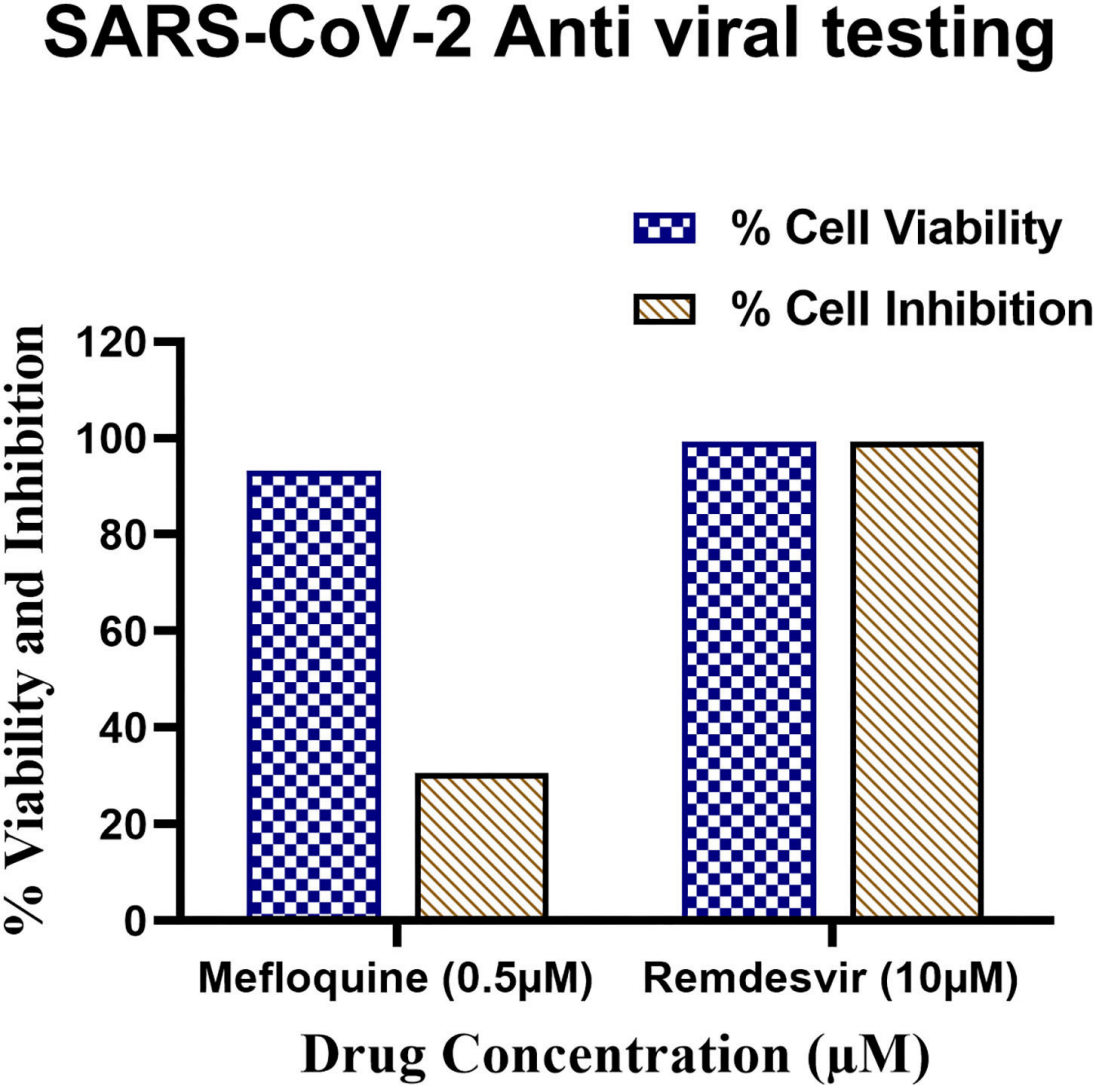
Antiviral testing of Mefloquine against SARS-CoV-2 in VeroE6 cells. (i) The ~10^4^ VeroE6 cells were pre-treated with Mefloquine and Remdesivir drug for 30 h at 37°C. Post-incubation, cells were stained with Hoechst 33342 and Sytox orange fluorescent dyes. The drug and control molecule treated cells were counted through stained Hoechst and Sytox image by using MetaXpress software. From raw images, the % average cell viability with control cells were calculated and the bar diagram highlighted in blue colour was plotted. (ii) 1 MOI of SARS-CoV-2 virus infected Vero E6 cells were treated with mefloquine drugs. The cells were fixed, permeabilized, and stained with mAb specific to SARS-CoV-2 nucleocapsid (primary) followed by alexafluor 568-labeled antibody (secondary) and all the cells were stained with Hoechst 33342 stain. The nucleocapsid positive and total nuclei cells were counted from the stained image and the % average cell inhibition with all the controls were calculated and the bar diagram highlighted in brown colour. All these assays were performed in triplicate wells.

The antiviral activity of a compound against SCoV-2 is measured in an immunofluorescence assay involving staining of virus-infected and drug-treated cells with SCoV-2 nucleocapsid (N)-specific mouse monoclonal antibody followed by incubation with Anti-mouse alexafluor 568 secondary antibody and then quantifying the fluorescence as a measure of SCoV-2 replication in Vero E6 cells. In these experiments, Remdesivir added at 10 μM concentration to SCoV-2-infected VeroE6 cells reduces SCoV-2 N-specific staining in >99% cells. Under these conditions, Mefloquine at 0.5 μM concentration was able to reduce the N-positivity in ~31% of SCoV-2 infected VeroE6 cells (Figure 10 and Supplementary Table 6). These results validated our claims about the antiviral properties of Mefloquine in HCoV-229E infected MRC-5 cells and also suggested it as a potential anti-SCoV-2 PLpro inhibitor drug for detailed investigations in future experiments.

## Discussion

### *In silico* Screening of FDA-Approved Drug Library

**Figure 11:**
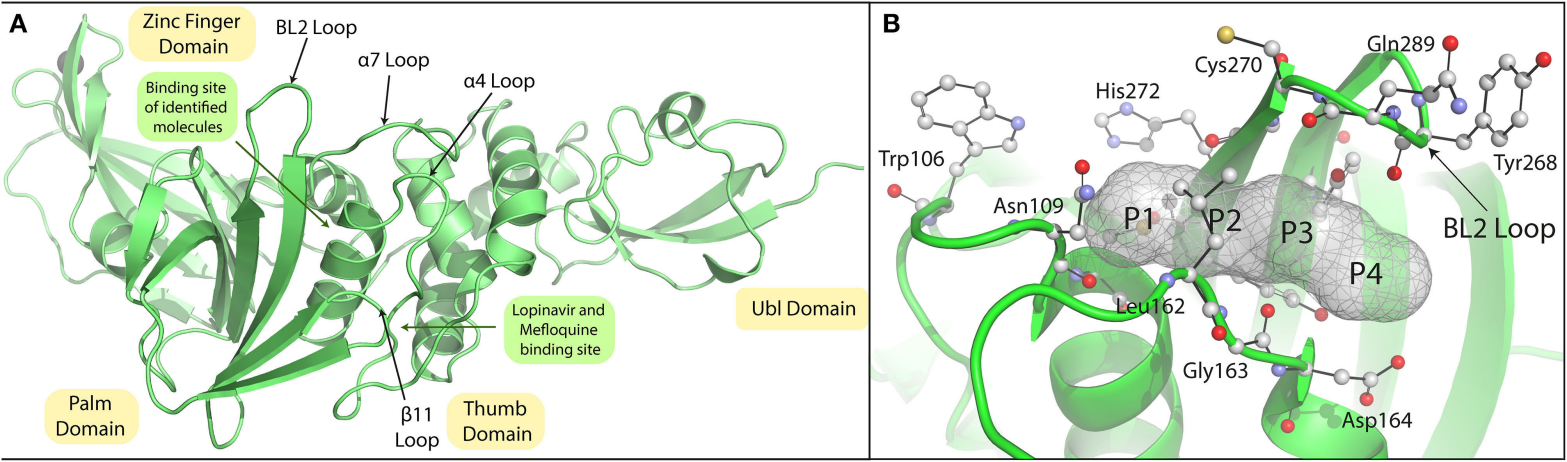
Crystal structure of SARS-CoV-2 PLpro and substrates pocket. **(A)** SARS-CoV-2 PLpro crystal structure (PDB ID:6WX4) highlighting the subdomains Ubl, Thumb, Zinc finger, and Palm domain highlighted in yellow colour. Active site loops are indicated by arrows. **(B)** P1-P4 positions of the peptide LRGG (PDB ID:4M0W) superimposed on SARS-CoV-2 PLpro (PDB ID:6WX4) are shown with mesh model and active site residues of these residues are shown with stick model.

The pandemic nature and continuous evolution of the virus to produce new variants of SCoV-2 with the ability to cause frequent outbreaks across the globe and the absence of effective vaccines against the new variants has necessitated the development of repurposed/novel anti-COVID therapeutics to contain the spread of existing SCoV-2 variants as well as to counter future variants. The PLpro of SCoV-2 is a crucial and multifunctional enzyme in the viral life cycle and is a well-established drug target. In the present study, we considered screening the FDA-approved drug library consisting of about 2,500 molecules to identify SCoV-2 PLpro inhibitors for drug-repurposing. Virtual screening was done using the SCoV-2 PLpro crystal structure (PDB ID: 6WX4), and through this screen a total of 12 potential hits were identified based on their binding energies and interactions with PLpro active site residues. Previous studies on SCoV-1 and MERS-CoV as well as the recent studies on SCoV-2 identified GRL0617 as a potential inhibitor of the PLpro. This drug has been shown to interact with the BL2 loop and occupy the S4 site blocking the entry of the substrate into the PLpro active site (Figure 11; Ratia et al., 2008). All the identified hits from the screening were docked into the PLpro, and were found to be bound with the catalytic site of the enzyme and many of them were interacting with the residues of the S1 subsite. A 100 ns MD simulation of the selected molecules with the SCoV-2 PLpro provided a vivid description of the protein-ligand interactions and their inhibition potential.

During the simulation, Mangafodipir, Glutathione disulfide, Ritonavir, FAD, Lopinavir, Mefloquine, and Rutin have shown interactions with the SCoV-2 PLpro active site residues through both polar and nonpolar interactions. Although binding to the catalytic site residues can directly interfere with the cleavage reaction, interaction with residues located elsewhere too can interfere with the catalytic cleavage by altering the conformation of the catalytic triad. All the selected molecules formed hydrogen bonds with the catalytic triad (Cys111, His272, and Asp286) and also formed stacking interactions with the His272 residue. Importantly, π-stacking interactions with Trp106 residue was a major contributor in the interactome for all these molecules. The Trp106 stabilizes the oxyanion hole formed in the transition state, therefore interaction with this residue can inhibit the catalytic efficiency of the enzyme (Ratia et al., 2008, 2014). Since the bicyclic rings of flavonoids and aromatic rings in the drugs effectively mediated the stacking interactions with the Trp106 residue, they may be considered essential while designing novel PLpro inhibitors. On the contrary, the polar regions of the molecules having a high number of hydrogen bond donors and acceptors were exposed to solvent as observed with Acarbose, Rutin, Darunavir, and Steviolbioside. The hydrogen bond between His272 and Asp286 is crucial for the functional positioning of His272 as it acts as a base for deprotonation of Cys111 during the catalytic cleavage (Ratia et al., 2008). The interaction of various drugs with PLpro lead to disruption of this hydrogen bond either by directly interacting with these residues or indirectly influencing the active site as observed in the case of FAD and Lopinavir. Steviolbioside, Iopromide, and Darunavir were unable to influence the native interaction between Asp286 and His272. Decomposition of the binding free energy was performed to identify the region of protein which energetically favours binding of Mefloquine (Supplementary Figure 3). The energy contributions highlight the residues Pro96, Ala288, Leu290, Ile285, Trp106, and Leu289. This shows the dominance of hydrophobic interactions in the stabilisation of Mefloquine and the nature of the pocket formed between the β11 loop and α7 loop. Interestingly, Mefloquine having the lowest TPSA value (45.15 Å^2^) had the most stable RMSD due to minimal interactions with solvent in contrast to drugs with higher TPSA values. Similar interactome was found for Lopinavir which had the second lowest TPSA value (120 Å^2^).

### *In vitro* PLpro-Inhibitory Activities of the Selected Compounds

We used recombinant bacterially expressed and chromatographically purified SCoV-2 PLpro in our protease-inhibition assays. In these assays, GRL0617 was found to inhibit PLpro activity with an IC_50_ value of ~198μM, which is ~100-fold higher compared to the reported value (2 μM). While it is apparent that the difference in IC_50_ values between the two studies is huge, this may be attributed to differences in assay conditions, like the enzyme-to-substrate concentrations, the quality, thereby the activity, of the recombinant enzyme, variations in buffer concentrations, etc. Normalisation of the IC_50_ data for the investigational drugs with that of GRL0617 suggested that they inhibited the PLpro activity at a relatively lower concentration range. However, here, we only reported the actual IC_50_ values without any normalizations for all the investigational drugs and GRL0617. Of the 9 compounds selected from the virtual screen, FAD hydrate and Mefloquine demonstrated satisfactory SCoV-2 PLpro inhibitory activity with IC_50_ values falling in the micromolar range. Flavin adenine dinucleotide (FAD) is a redox cofactor of several important reactions in the body metabolism (Schnekenburger and Diederich, 2015). Although there is no record of a direct antiviral role, it has been previously reported to potentiate the antiviral activity of interferons and other antiviral molecules (Jamison et al., 1990; Saito et al., 1996). On the other hand, Mefloquine is an anti-malarial drug with an established activity against the malarial parasite during its life cycle in human red blood cells. With regard to its antiviral activities, Mefloquine has been shown to be effective against Feline calicivirus and Feline coronavirus, and more recently it has also been shown to be effective against SCoV-2, where it was found to target the viral spike protein and act as an entry inhibitor in a prophylactic role (McDonagh et al., 2015; Izes et al., 2020; Shionoya et al., 2021). On the other hand, Mefloquine, an anti-malarial drug, only moderately (IC_50_ = 459 μM) inhibited the SCoV-2 PLpro activity *in vitro* in our enzyme-inhibition assays.

Lopinavir and Ritonavir are protease inhibitors and are used in combination in the treatment of AIDS to inhibit HIV protease activity. Supporting the role of these drugs in COVID-19, some of the studies demonstrated binding of Lopinavir/Ritonavir with SCoV-2 3CLpro through *in-silico* studies. Notably, these studies demonstrated higher affinity of Ritonavir for the SCoV-2 3CLpro compared to Lopinavir (Nutho et al., 2020). However, there is no evidence to suggest the inhibitory role of Lopinavir/Ritonavir against SCoV-2 PLpro. In the present study, Lopinavir and Ritonavir demonstrated moderate to weak inhibitory activity against SCoV-2 PLpro with an IC_50_ value of 1.0 ±.1 mM and 1.0 ±.32 mM, respectively, probably suggesting weaker interaction with the SCoV-2 PLpro.

### Antiviral Activity of Mefloquine

Among the 9 investigational drugs, Mefloquine and Lopinavir (please refer to Section Antiviral Activities of Other Investigational Drugs Against HCoV-229E) demonstrated superior antiviral activities against HCoV-229E in MRC-5 cells. Accordingly, Mefloquine was able to protect ~60% of HCoV-229E-infected MRC-5 cells from virus-induced CPE when treated with 120 μM Mefloquine. Though both Mefloquine and Lopinavir demonstrated superior activities against HCoV-229E, we chose only Mefloquine to further investigate its anti-SCoV-2 activity because of logistical reasons as well as due to the prior availability of clinical data on Lopinavir in COVID patients (Cao et al., 2020a,b). Accordingly, we tested the antiviral activity of Mefloquine against SCoV-2 in Vero E6 cells. Validating our hypothesis that the PLpro inhibitors against one coronavirus would also inhibit that of other coronaviruses in a similar manner, Mefloquine that demonstrated maximum antiviral activity (60% inhibition) against HCoV-229E at 120 μM concentration in infected MRC-5 cells also inhibited SCoV-2 replication (30% inhibition) at the only assayed concentration of 500 nM in infected Vero E6 cells.

Corroborating our finding with Mefloquine in a therapeutic role, other recent studies demonstrated similar antiviral activity of Mefloquine against SCoV-2 (Fan et al., 2020; Gendrot et al., 2020; Jeon et al., 2020; Weston et al., 2020; Shionoya et al., 2021). But, it should be noted that none of these studies investigated the mechanism of action or the molecular target for Mefloquine. While Shionoya et al. found a prophylactic role for Mefloquine against SCoV-2, we have demonstrated a therapeutic role for Mefloquine possibly through its protease inhibitory activity against viral PLpro (Shionoya et al., 2021). Further, by targeting PLpro, Mefloquine is not only hindering the polyprotein hydrolysis but is also helping in host antiviral responses by inhibiting PLpro-mediated suppression of host immune responses (Rut et al., 2020). Mefloquine is an anti-malarial drug with an established activity against the malarial parasite during its life cycle in human red blood cells. This drug is contraindicated in persons with a history of psychiatric disorders and in those who are hypersensitive to the drug. While a few adverse events are reported among Mefloquine users, these are rare and the risk stratification for these events can be performed based on medical history, physical examination, and with the available investigations. A recent study opined that a cumulative dose of 24 mg/kg given over a period of 3 days at 8 mg/kg is well tolerated and effective against malaria (Lee et al., 2017).

Nevertheless, as an antiviral candidate, Mefloquine has been shown to be effective against feline calicivirus and feline coronavirus, and more recently it has also been shown to be effective against SCoV-2, where it was found to target the viral spike protein and act as an entry inhibitor in a prophylactic role (McDonagh et al., 2015; Izes et al., 2020; Shionoya et al., 2021). Based on the findings in our study and from other studies, we opine that Mefloquine is a potential interventional drug and therefore needs to be studied in more detail in future preclinical and clinical studies. If found to be effective both as a prophylactic and as a therapeutic intervention, due to relatively fewer and rare adverse events, ease of risk stratification and monitoring, Mefloquine will have its uses both during the early stages of COVID as a prophylactic as well as in hospitalized patients with severe COVID in a therapeutic role.

### Antiviral Activities of Other Investigational Drugs Against HCoV-229E

Besides Mefloquine, Lopinavir demonstrated superior antiviral activities against HCoV-229E in MRC-5 cells. Accordingly, treatment with 40 μM Lopinavir resulted in superior antiviral activity against HCoV-229E in cell-based assays protecting ~75% of HCoV-229E-infected MRC-5 cells from virus-induced CPE, which effectively translated into ~1.4 log_10_ reduction in viral load (*R* = −0.8629, *p* = 0.0269), whereas treatment with 70 μM Ritonavir imparted only about 35% protection from virus-induced CPE in MRC-5 cells, which resulted in ~0.6 log_10_ reduction in viral load (*R* = −0.9476, *p* = 0.0012). So, together, we hypothesize that the antiviral activities observed for Lopinavir and Ritonavir were actually due to their inhibitory activity against HCoV-229E PLpro rather than against the HCoV-229E 3CLpro.

Though both Ritonavir and Lopinavir are protease inhibitors, Ritonavir is generally used to inhibit cytochrome P450 3A4 so as to increase the plasma availability of Lopinavir during antiretroviral therapy in HIV patients. With regard to their role in COVID-19 treatment, encouraged by their activity against SCoV-1 in earlier trials, the combination was tested in a randomized, controlled, open-label trial involving 199 adult patients hospitalized for severe confirmed COVID-19, where the study authors concluded them ineffective (Cao et al., 2020a). However, after several research groups contested the methodology used and the way the data was analysed, authors of the clinical trial reconsidered their original conclusions on the study outcome and declared the combination treatment could potentially offer clinical benefit against COVID-19 (Cao et al., 2020a). In our *in vitro* study, treatment with 40 μM Lopinavir protected ~75% of HCoV-229E-infected MRC-5 cells from virus-induced CPE, which effectively translated into ~1.4 log_10_ reduction in viral load (*R* = −0.8629, *p* = 0.0269), whereas treatment with 70 μM Ritonavir imparted only about 35% protection from virus-induced CPE in MRC-5 cells and resulted in ~0.6 log_10_ reduction in viral load (*R* = −0.9476, *p* = 0.0012).

Antiviral activity demonstrated by Darunavir, another antiretroviral drug, against the HCoV-229E was similar (~35% of virus-infected cells protected from virus-induced CPE) to that was achieved with Ritonavir, but it translated into a higher (1.42 log_10_ for Darunavir) reduction in viral load. Interestingly, a recent *in vitro* study found that Darunavir was completely ineffective against SCoV-2 at clinically relevant concentrations (Meyer et al., 2020). Though the drug interacted with SCoV-2 PLpro, the interactions with the active site were weak and insignificant (Cao et al., 2020a). Similar to Ritonavir and Darunavir, Rutin trihydrate was able to protect about 37% of virus-infected cells from virus-induced CPE, but this effect translated into only about 0.2 log_10_ reduction in viral titers. Rutin is a dietary flavonoid and has received a great attention due to its range of pharmacological attributes, including antimicrobial properties. Notably, Rutin has been shown to interfere with the cell entry of Hepatitis C virus into hepatoma cells (Bose et al., 2017). Here, in the present study, Rutin was tested in a therapeutic mode, which may be the reason for its weaker effects on viral load. Accordingly, future studies should aim at investigating the prophylactic activity of Rutin to exploit its true anti-CoV potential.

On the other hand, though FAD hydrate demonstrated superior inhibitory activity against SCoV-2 PLpro with an IC_50_ value of 127 μM, which was better than that of GRL0617, FAD hydrate failed to show any significant antiviral activity against HCoV-229E when tested in virus-infected cell culture model, while GRL0617 was found to be only moderately effective. This can be explained by differences in the drug uptake and its intracellular kinetics. Accordingly, it is possible that FAD hydrate was poorly taken up by MRC-5 cells and/or very little concentration of the drug was available at the site of action during viral replication, which probably contributed for the poor *in vitro* effects of FAD hydrate in virus-infected MRC-5 cells.

### HCoV-229E as a Suitable Surrogate for SCoV-2 Drug Discovery

In the present study, we used HCoV-229E as a surrogate virus for SCoV-2 to demonstrate the antiviral effects of the selected FDA-approved compounds. Though the PLpro of HCoV-229E and SCoV-2 shared only about 22% identity and about 53% similarity at the amino acid level, they are very similar at the structural level. Importantly, *in silico* analysis showed five out of six amino acids in the catalytic site of PLpro are conserved, except for the Tryptophan-to-Threonine at residue 106 (W106T), with total ~86% similarity (Supplementary Figure 4). Further, docking of the drugs against HCoV-229E PLpro suggested similarities in binding patterns and drug-protein interactions with those observed with the SCoV-2 PLpro. Probably validating these findings, Mefloquine that inhibited HCoV-229E replication *in vitro* in infected MRC-5 cells also inhibited SCoV-2 replication in infected Vero E6 cells. To further demonstrate the similarities between the two viruses, we compared their genomes and the signalling pathways involved in disease pathogenesis and host immune responses. We found a comparable level of similarity between the two genomes as well as in various pathways involved in pathogenesis and antiviral responses. Accordingly, the published literature suggests that the IFN-stimulated genes (ISGs) inhibit the infections with the two viruses in a similar fashion through overlapping pathways (Zhao et al., 2022). Supporting our hypothesis, some of the recent studies demonstrated the similarities in antiviral activities of pharmaceutical compounds against HCoV-229E and SCoV-2 (Siddell et al., 1983; Masters, 2006; Weiss and Leibowitz, 2011). Further, the molecular mechanisms involved in disease pathogenesis, particularly those involved in cytokine storm, inflammation, and stress from a cell biology point of view are mostly similar. Overall, the overlap in antiviral networks and signalling pathways involved in disease pathogenesis together with the demonstration of overlapping *in silico* data for the selected compounds with PLpro from SCoV-2 and the HCoV-229E, and of the antiviral activities of Mefloquine in cell culture models of HCoV-229E and SCoV-2 justify the use of HCoV-229E as a surrogate for SCoV-2 in the present study.

## Conclusion

Virtual screening is an effective approach to identify potential drugs for further development through laboratory and clinical investigations. Since, FDA-approved clinical drugs are field-tested for safety and effectiveness, they are well-suited for expedited clinical development in a cost effective manner as repurposed drugs for COVID-19 treatment. Due to its critical roles during viral replication, we screened the FDA-approved drug library, and identified 12 potential molecules. Further, using MM-PBSA method we established their molecular interactions with SCoV-2 PLpro through MD simulation studies and also estimated their binding free energy. From these *in-silico* studies, we further shortlisted 9 drugs for *in vitro* biochemical and cell culture-based studies. Their affinity for SCoV-2 PLpro was investigated using bacterially expressed PLpro in a biochemical protease-inhibition assay using single peptide-AMC fluorogenic probe as a substrate. Validating the conformity of our assay system with the published work, GRL0617, a well-established inhibitor of SCoV-2 PLpro activity and a reference control in our assays, demonstrated PLpro-inhibitory activity with an IC_50_ value of ~198μM. Among the selected FDA-approved drugs, we identified FAD (IC_50_ value ~127 μM) and Mefloquine (IC_50_ value~459 μM) as potential inhibitors of SCoV-2 PLpro. Attributable to their structural similarities in the catalytic core of PLpro and similarities in post-entry replication mechanisms, we used HCoV-229E as a surrogate model for SCoV-2 and tested the 9 selected molecules for antiviral activities in cell culture systems. Consistent with our *in vitro* protease-inhibition assays using SCoV-2 PLpro, Mefloquine demonstrated superior antiviral effects against HCoV-229E compared to GRL0617. Though Lopinavir moderately inhibited PLpro activity compared to Mefloquine and GRL0617 in protease-inhibition experiments, its antiviral activity against HCoV-229E was better than that of other selected FDA-approved drugs. Since the focus of the present study was to identify a suitable PLpro inhibitor which will inhibit the SCoV2 replication, we tested Mefloquine against the SCoV-2 in Vero E6 cells, where it was found to be potent against SCoV-2 as well. While we could not investigate dose-dependent effects of Mefloquine on SCoV-2 majorly due to logistic issues, we propose Mefloquine as a potential PLpro inhibitor for further development in preclinical studies and, if found to be effective, in clinical studies as well. Together, based on the reported similarities in disease pathogenesis and antiviral pathways combined with the demonstration of similar inhibitory effects of potential antiviral compounds on both HCoV-229E and SCoV-2 across studies, we propose HCoV-229E as a suitable surrogate for SCoV-2 in drug-discovery studies, particularly aimed at identifying potential inhibitors of post-entry events during virus replication.

## Data Availability Statement

The original contributions presented in the study are included in the article/Supplementary Material, further inquiries can be directed to the corresponding author/s.

## Author Contributions

RK, MKa, and KI: conceptualisation. RK, TK, AD, VK, and MA: methodology. AS, DS, KB, TS, and SKa: software. RK, TK, AP, MA, MKa, and KI: validation and formal analysis. RK, TK, AD, and MA: investigation and data curation. SG, KP, and PJ: resources. RK, MA, MKa, and KI: writing-original and draft and visualization. RK, TK, VK, PS, MKu, SKu, AP, DS, MA, MKa, and KI: writing—review and editing. MKa and KI: supervision and publication funding acquisition. KI: project administration and funding acquisition. All authors have read and agreed to the published version of the manuscript.

## Funding

This work was financially supported by grants from AIIMS (Ref # A-COVID-4), DST_SERB (Ref # EEQ/2016/000507), DST_SERB (Ref # CRG/2019/003546), ICMR (Ref # ISRM/12(39)/2019), and DBT (Ref # BT/PR34319/Med/29/1488/2019). Also, MA, Srikara Biologicals Pvt., Ltd., is thankful to BIRAC for supporting the studies on HCoV-229E under the COVID consortium (Ref # BT/COVID0035/01/20).

## Conflict of Interest

MA and SKa were commercial employed by Srikara Biologicals Private Limited, Tirupati. The remaining authors declare that the research was conducted in the absence of any commercial or financial relationship that could be construed as a potential conflict of interest.

## Publisher's Note

All claims expressed in this article are solely those of the authors and do not necessarily represent those of their affiliated organizations, or those of the publisher, the editors and the reviewers. Any product that may be evaluated in this article, or claim that may be made by its manufacturer, is not guaranteed or endorsed by the publisher.

## Abbreviations

AMC: 7-Amino-4-methylcoumarin
COVID-19: Coronavirus Disease 2019
CPE: Cytopathic Effects
FDA: Food and Drug Administration
GRL0617: (R)-5-amino-2-methyl-N-(1-(naphthalen-1-yl)ethyl) benzamide (3)
HCoV-229E: Human Coronavirus 229E strain
MERS-CoV: Middle East Respiratory Syndrome Coronavirus
MD: Molecular Dynamics
MM-PBSA: Molecular Mechanics Poisson–Boltzmann Surface Area
PLpro: Papain-Like protease
RMSD: Root Mean Square Deviation
SARS-CoV-2/SCoV-2: Severe Acute Respiratory Syndrome Corona Virus 2.
