# Supplementary material for: Repurposing of FDA Approved Drugs Against SARS-CoV-2 Papain-Like Protease: Computational, Biochemical, and in vitro Studies
Source: Front Microbiol. 2022 May 10;13:877813. doi: 10.3389/fmicb.2022.877813 (PMC9127501; doi:10.3389/fmicb.2022.877813)
Supplement: Supplementary file 1 [file Table_1.DOCX]

**Supplementary Information for publications**

**Supplementary Figure 1:** **(A)** Backbone and **(B)** ligand RMSD plots for the 100ns molecular dynamics trajectory of the 12 docked complexes. No significant change on the backbone protein structure was observed in the backbone RMSD plot. Relative ligand stability was inferred from the ligand RMSD plot.


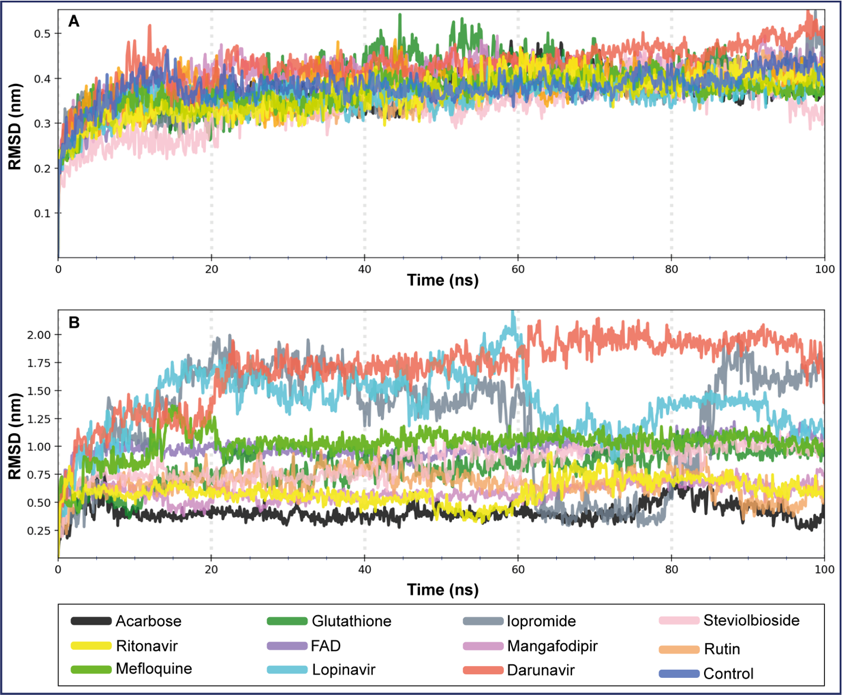


**Supplementary Figure 2:** Parameters obtained from MD simulations of PLpro with the screened molecules **(A)** RMSF plot **(B)** Radius of Gyration plot and for the 100ns MD simulations.


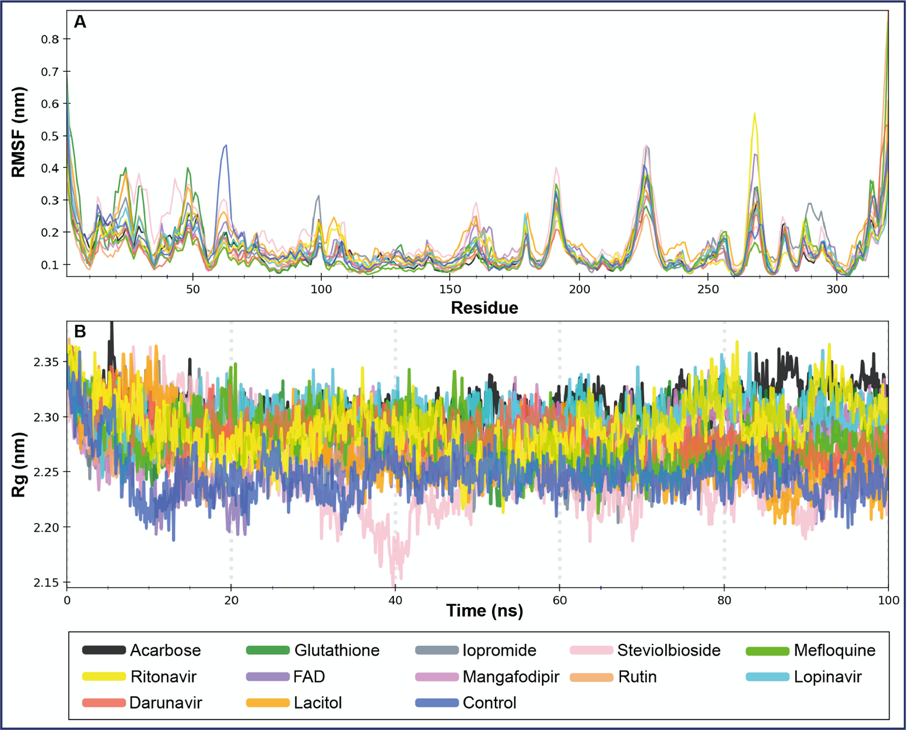


**Supplementary Figure 3: (A)** Binding free energy for the identified molecules computed using MMPBSA method **(B)** Per-residue decomposition of the binding free energy for Mefloquine and Lopinavir.


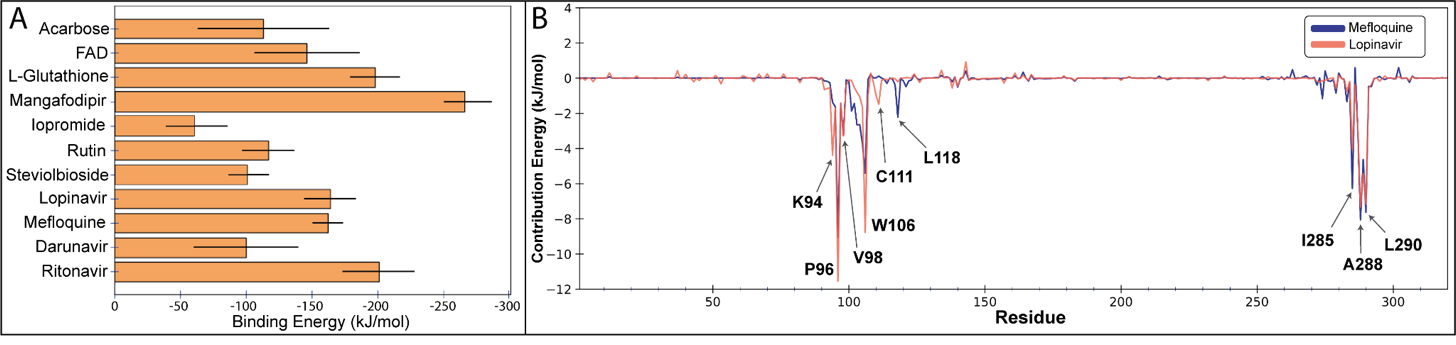


**Supplementary Figure 4:** Structural comparison of HCoV-229E PLpro and SARS-CoV-2 PLpro. **(A)** Comparison between HCoV-229E PLpro modelled structure (shown in orange) and SARS-CoV-2 PLpro crystal structure (shown in green). **(B)**  Active site of the modelled HCoV-229E PLpro (orange) and SARS-CoV-2 PLpro (green) catalytic active site.


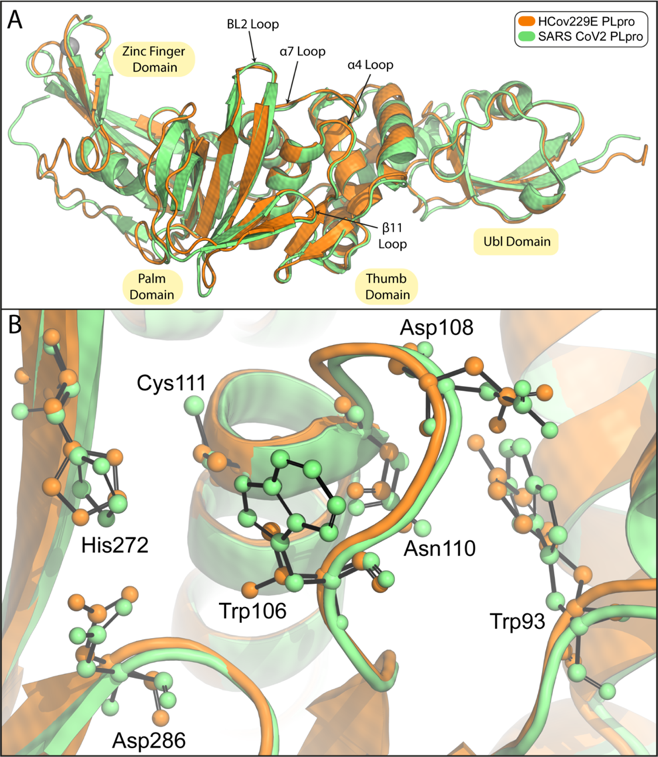


**Supplementary Figure 5 :** ^1^H NMR of (*R*)-5-amino-2-methyl-N-(1-(naphthalen-1-yl)ethyl)benzamide **3.** The illustrated proton chemical shift values confirm the formation of GRL-0167 which is further supported by ^13^C and ^1^H-^1^H COSY NMR.


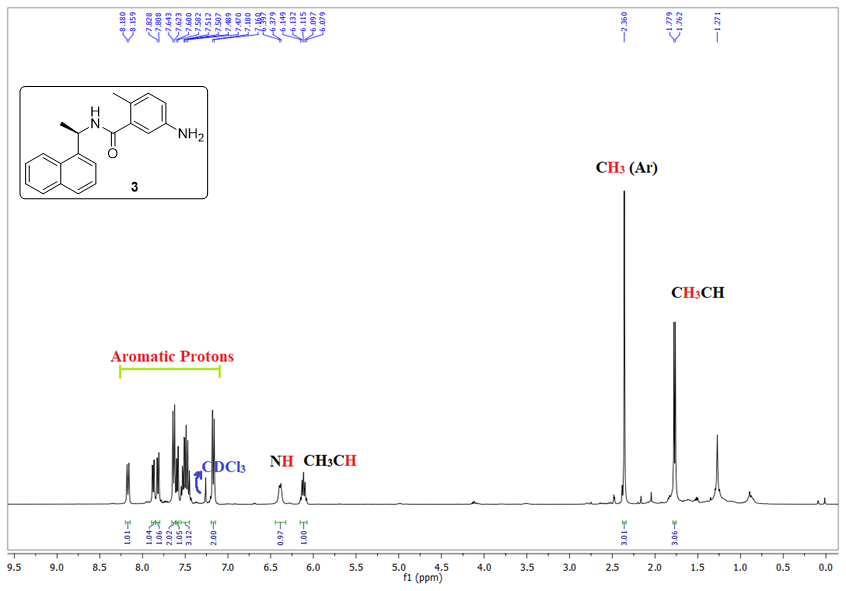


**Supplementary Figure 6 :** ^13^C NMR (100 MHz, CDCl_3_) of (*R*)-5-amino-2-methyl-N-(1-(naphthalen-1-yl)ethyl)benzamide **3**. The chemical shift *δ* 166.3 corresponds to the carbonyl group of amide bond and all other chemical shifts illustrated in detail, confirms the formation of GRL-0617.

**
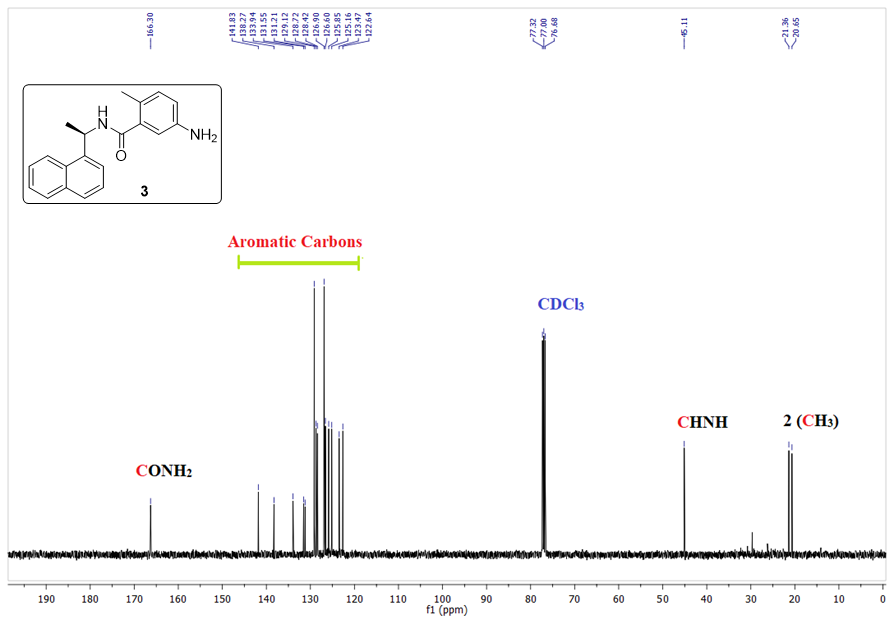
**

**Supplementary Figure 7:** ^1^H-^1^H COSY (400 MHz, CDCl_3_) of (*R*)-5-amino-2-methyl-N-(1-(naphthalen-1-yl)ethyl)benzamide 3. The coupling between N***H*** and C***H***CH_3_ and C***H***_3_CHNH and C***H***CH_3_ along with ^1^H and ^13^C NMR confirms the formation of GRL-0617.

**
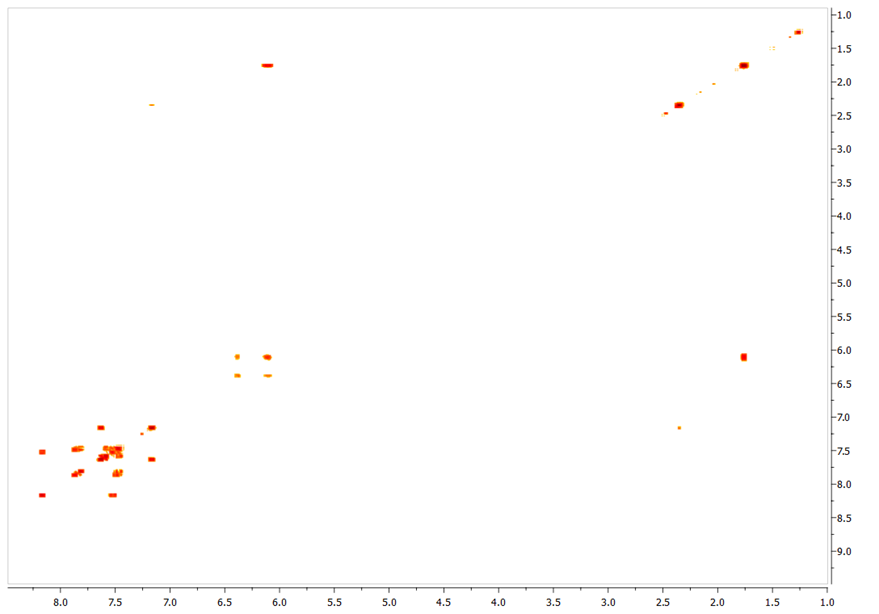
**

**Supplementary Information 8:** SARS-CoV-2 PLpro DNA sequence was codon-optimized for bacterial expression system and the recombinant constructs in pET22b were used in the study for production of recombinant SARS-CoV-2 PLpro used in protease-inhibition assays *in vitro*.

**SARS-CoV-2 PLpro DNA codon optimized Sequence in *E.coli***

GAAGTGCGTACCATCAAAGTGTTCACCACCGTTGATAACATTAACCTGCACACCCAGGTGGTTGACATGAGCATGACCTACGGTCAGCAATTTGGTCCGACCTATCTGGACGGCGCGGATGTTACCAAGATTAAACCGCACAACAGCCACGAAGGTAAAACCTTCTACGTGCTGCCGAACGACGATACCCTGCGTGTTGAGGCGTTCGAATACTATCACACCACCGACCCGAGCTTTCTGGGCCGTTACATGAGCGCGCTGAACCACACCAAGAAATGGAAGTATCCGCAAGTTAACGGTCTGACCAGCATCAAATGGGCGGATAACAACTGCTATCTGGCGACCGCGCTGCTGACCCTGCAACAAATTGAGCTGAAGTTCAACCCGCCGGCGCTGCAAGACGCGTACTATCGTGCGCGTGCGGGCGAAGCGGCGAACTTTTGCGCGCTGATCCTGGCGTACTGCAACAAAACCGTGGGCGAGCTGGGCGATGTTCGTGAAACCATGAGCTATCTGTTCCAACACGCGAACCTGGACAGCTGCAAGCGTGTGCTGAACGTGGTTTGCAAAACCTGCGGTCAGCAACAGACCACCCTGAAGGGCGTGGAGGCGGTTATGTACATGGGCACCCTGAGCTATGAACAATTTAAGAAAGGCGTTCAGATTCCGTGCACCTGCGGTAAACAGGCGACCAAATACCTGGTGCAACAGGAGAGCCCGTTCGTTATGATGAGCGCTCCGCCGGCGCAATATGAGCTGAAACACGGCACCTTTACCTGCGCGAGCGAATACACCGGTAACTATCAGTGCGGCCACTACAAGCACATCACCAGCAAAGAAACCCTGTATTGCATTGATGGCGCGCTGCTGACCAAGAGCAGCGAATACAAAGGTCCGATCACCGACGTGTTTTACAAAGAGAACAGCTACACCACCACCATCAAACCCGGGCACCATCATCACCACCACTAA

N-terminal 6X his-tag with SUMO tag, SARS-CoV-2_PLpro optimized DNA sequence with C-terminal 6XHis tag

ATGCACCACCACCACCACCATATGGACAGCGAAGTGAATCAGGAAGCGAAACCGGAAGTTAAGCCGGAAGTGAAACCGGAAACCCACATCAACCTGAAGGTGAGCGATGGTAGCAGCGAAATCTTCTTCAAAATTAAGAAAACCACCCCGCTGCGTCGTCTGATGGAGGCGTTCGCGAAGCGTCAGGGCAAAGAAATGGACAGCCTGACCTTTCTGTACGATGGTATCGAGATTCAAGCGGACCAGACCCCGGAGGACCTGGATATGGAAGACAACGACATCATTGAGGCGCACCGTGAACAAATCGGTGGCCTGAAGGGTGGCGCTAGCGAAGTGCGTACCATCAAAGTGTTCACCACCGTTGATAACATTAACCTGCACACCCAGGTGGTTGACATGAGCATGACCTACGGTCAGCAATTTGGTCCGACCTATCTGGACGGCGCGGATGTTACCAAGATTAAACCGCACAACAGCCACGAAGGTAAAACCTTCTACGTGCTGCCGAACGACGATACCCTGCGTGTTGAGGCGTTCGAATACTATCACACCACCGACCCGAGCTTTCTGGGCCGTTACATGAGCGCGCTGAACCACACCAAGAAATGGAAGTATCCGCAAGTTAACGGTCTGACCAGCATCAAATGGGCGGATAACAACTGCTATCTGGCGACCGCGCTGCTGACCCTGCAACAAATTGAGCTGAAGTTCAACCCGCCGGCGCTGCAAGACGCGTACTATCGTGCGCGTGCGGGCGAAGCGGCGAACTTTTGCGCGCTGATCCTGGCGTACTGCAACAAAACCGTGGGCGAGCTGGGCGATGTTCGTGAAACCATGAGCTATCTGTTCCAACACGCGAACCTGGACAGCTGCAAGCGTGTGCTGAACGTGGTTTGCAAAACCTGCGGTCAGCAACAGACCACCCTGAAGGGCGTGGAGGCGGTTATGTACATGGGCACCCTGAGCTATGAACAATTTAAGAAAGGCGTTCAGATTCCGTGCACCTGCGGTAAACAGGCGACCAAATACCTGGTGCAACAGGAGAGCCCGTTCGTTATGATGAGCGCTCCGCCGGCGCAATATGAGCTGAAACACGGCACCTTTACCTGCGCGAGCGAATACACCGGTAACTATCAGTGCGGCCACTACAAGCACATCACCAGCAAAGAAACCCTGTATTGCATTGATGGCGCGCTGCTGACCAAGAGCAGCGAATACAAAGGTCCGATCACCGACGTGTTTTACAAAGAGAACAGCTACACCACCACCATCAAACCCGGGCACCATCATCACCACCACTAA

**SARS-CoV-2 PLpro Codon optimized(Auto cleavable N-terminal 6X his-tag with SUMO tag and Uncleavable C-terminal 6X His tag)-protein sequence:**

MHHHHHHMDSEVNQEAKPEVKPEVKPETHINLKVSDGSSEIFFKIKKTTPLRRLMEAFAKRQGKEMDSLTFLYDGIEIQADQTPEDLDMEDNDIIEAHREQIGGLKGGASEVRTIKVFTTVDNINLHTQVVDMSMTYGQQFGPTYLDGADVTKIKPHNSHEGKTFYVLPNDDTLRVEAFEYYHTTDPSFLGRYMSALNHTKKWKYPQVNGLTSIKWADNNCYLATALLTLQQIELKFNPPALQDAYYRARAGEAANFCALILAYCNKTVGELGDVRETMSYLFQHANLDSCKRVLNVVCKTCGQQQTTLKGVEAVMYMGTLSYEQFKKGVQIPCTCGKQATKYLVQQESPFVMMSAPPAQYELKHGTFTCASEYTGNYQCGHYKHITSKETLYCIDGALLTKSSEYKGPITDVFYKENSYTTTIKPGHHHHHH-

**SARS-CoV-2 PLpro Codon optimized(Uncleavable C-terminal 6X His tag)-protein sequence:**

EVRTIKVFTTVDNINLHTQVVDMSMTYGQQFGPTYLDGADVTKIKPHNSHEGKTFYVLPNDDTLRVEAFEYYHTTDPSFLGRYMSALNHTKKWKYPQVNGLTSIKWADNNCYLATALLTLQQIELKFNPPALQDAYYRARAGEAANFCALILAYCNKTVGELGDVRETMSYLFQHANLDSCKRVLNVVCKTCGQQQTTLKGVEAVMYMGTLSYEQFKKGVQIPCTCGKQATKYLVQQESPFVMMSAPPAQYELKHGTFTCASEYTGNYQCGHYKHITSKETLYCIDGALLTKSSEYKGPITDVFYKENSYTTTIKPGHHHHHH-
