# Supplementary material for: Repurposing of FDA Approved Drugs Against SARS-CoV-2 Papain-Like Protease: Computational, Biochemical, and in vitro Studies
Source: Front Microbiol. 2022 May 10;13:877813. doi: 10.3389/fmicb.2022.877813 (PMC9127501; doi:10.3389/fmicb.2022.877813)
Supplement: Supplementary file 2 [file Table_2.DOCX]

**Supplementary Information Table 1:** Average backbone and ligand RMSD values observed during 100ns MD simulations

| **Molecule** | **Average Backbone RMSD (nm)** | **Standard Deviation (nm)** | **Average Ligand RMSD(nm)** | **Standard Deviation (nm)** |
| --- | --- | --- | --- | --- |
| Acarbose | 0.37 | 0.04 | 0.41 | 0.08 |
| FAD | 0.37 | 0.04 | 0.99 | 0.09 |
| Glutathione Disulfide | 0.39 | 0.06 | 0.81 | 0.16 |
| Mangafodipir | 0.41 | 0.04 | 0.59 | 0.08 |
| Iopromide | 0.36 | 0.04 | 1.24 | 0.50 |
| Rutin | 0.36 | 0.04 | 0.67 | 0.11 |
| Steviolbioside | 0.34 | 0.06 | 0.85 | 0.15 |
| Lactitol | 0.38 | 0.04 | 10.73 | 5.83 |
| Lopinavir | 0.36 | 0.04 | 1.39 | 0.28 |
| Mefloquine | 0.37 | 0.04 | 1.02 | 0.13 |
| Darunavir | 0.43 | 0.05 | 1.68 | 0.31 |
| Ritonavir | 0.36 | 0.05 | 0.60 | 0.11 |
| Control | 0.38 | 0.03 | - | - |

**Supplementary Information Table 2:** List of interacting residues observed during the 100ns simulations of PLpro-drug complexes

| **S. No.** | **Database ID** | **Generic Name** | **Hydrogen-bond Interactions** | **Hydrophobic Interactions** |
| --- | --- | --- | --- | --- |
| 1. | DB00284 | Acarbose | Asp286 (113%), Ala288 (65%), Tyr273 (75%), Trp106(60%), Gly271 (47%) | - |
| 2. | DB03310 | Glutathione Disulphide | Asp108 (31%), His272 (21%), Gly271 (15%), Gln269 (14%), Cys270 (13%) | Trp106, His272 |
| 3. | DB06796 | Mangafodipir | Asp286(26%), Cys111(33%), Tyr268 (11%) | Trp106, His272, Ala107, Tyr112, Ile104 |
| 4. | DB03147 | FAD | Asp108(180%), Trp106 (62%), Lys93 (49%), Lys94 (41%), Lys92 (Ionic Interaction) | Trp106, Trp93, Ala107 |
| 5. | DB09156 | Iopromide | Asp108 (63%) | - |
| 6. | DB01698 | Rutin | Asp286 (78%), Asn109 (42%), Asp108 (38%), Ala107 (11%) | Trp106, His272, Ala107 |
| 7. | DB012434 | Steviolbioside | Asp288 (83%), Asp108 (77%), Trp106 (43%) | Leu289, Pro96, Ala107, Ala288 |
| 8. | DB012942 | Lactitol | - | - |
| 9. | DB01601 | Lopinavir | Leu290 (27%),Ala288 (16%), Gln97 (12%) | Pro96, Val98, Trp106, Tyr283, Ile285, Ala288, Leu289, Leu290 |
| 10. | DB00358 | Mefloquine | Gly287 (55%), Trp106 (14%) | Pro96, Val98, Leu101, Ile104. Trp106, Leu118, His272, Tyr283, Ile285, Ala288, Leu289, Leu290, |
| 11. | DB01264 | Darunavir | Gln269 (25%), Gly160 (23%), Cys270 (21%), Glu161 (11%) | Leu162, Ala107 |
| 12. | DB00503 | Ritonavir | Asn109 (55%), Asp108(42%), Ala107 (13%) | Trp106, His272, Ala107, Tyr112, Leu162, Ile285 |

**Supplementary Information Table 3:** Energy values obtained from MMPBSA calculations after MD simulation

| **Molecule Name** | **van der  Waal energy**  **(kJ/mol)** | **+/-** | **Electrostatic energy**  **(kJ/mol)** | **+/-** | **Polar  solvation  energy**  **(kJ/mol)** | **+/-** | **SASA energy**  **(kJ/mol)** | **+/-** | **Binding energy**  **(kJ/mol)** | **+/-** |
| --- | --- | --- | --- | --- | --- | --- | --- | --- | --- | --- |
| Acarbose | -196.672 | 20.77 | -52.899 | 24.746 | 153.848 | 50.318 | -17.366 | 2.269 | -113.089 | 50.2 |
| FAD | -223.511 | 20.305 | -70.076 | 28.098 | 165.268 | 44.258 | -18.018 | 2.39 | -146.336 | 40.189 |
| Glutathione Disulfide | -252.308 | 23.476 | -48.581 | 20.891 | 121.513 | 28.599 | -18.968 | 1.712 | -198.343 | 18.959 |
| Rutin | -169.612 | 18.361 | -59.766 | 25.799 | 127.010 | 26.955 | -14.446 | 1.444 | -116.814 | 19.887 |
| Lopinavir | -199.473 | 17.865 | -21.460 | 8.713 | 75.451 | 15.332 | -18.563 | 1.744 | -164.045 | 19.599 |
| Mefloquine | -181.664 | 10.888 | -10.475 | 6.312 | 45.050 | 6.308 | -15.284 | 0.976 | -162.373 | 11.599 |
| Darunavir | -137.582 | 16.308 | -44.211 | 19.076 | 93.596 | 37.403 | -11.754 | 1.957 | -99.952 | 39.833 |
| Ritonavir | -264.926 | 25.964 | -6.706 | 8.125 | 92.608 | 15.647 | -21.920 | 1.630 | -200.944 | 27.564 |
| Iopromide | -69.601 | 20.207 | -32.723 | 23.711 | 51.536 | 32.884 | -9.359 | 2.519 | -60.147 | 25.482 |
| Mangafodipir | -347.260 | 22.534 | -25.401 | 11.392 | 130.473 | 11.017 | -23.461 | 1.167 | -265.648 | 21.536 |
| Steviolbioside | -141.709 | 15.502 | -48.852 | 25.387 | 103.872 | 29.227 | -13.537 | 1.738 | -100.227 | 17.041 |
| Lactitol | - | - | - | - | - | - | - | - | - | - |

**Supplementary Information Table 4:** List of selected 12 FDA approved drugs docked against HCoV-229E PLpro

| **S. No.** | **Database ID** | **Generic Name** | **2D structures** | **Docking Score** | **Glide Energy** | **H-bond interactions** | **Ionic Interactions** | **Hydrophobic Interactions** |
| --- | --- | --- | --- | --- | --- | --- | --- | --- |
| 1. | DB00284 | Acarbose | 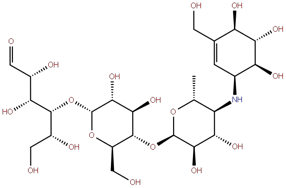 | -9.382 | -44.765 | Thr100, Asp102, Asn 103, His267, Asp280 | Lys99, Lys153, Lys156, Lys265, His267, Arg283 | Asp102 |
| 2. | DB03310 | Glutathione Disulphide | 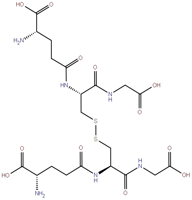 | -5.622 | -47.136 | Thr100,Asp102, Asn103, His267 | Lys99, Lys153, Lys156, Lys265, His267 | - |
| 3. | DB06796 | Mangafodipir | 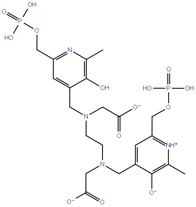 | -6.676 | -46.410 | Thr100, Asp102, Asn103, Lys156, Lys265, His267 | Lys156, Arg283 | - |
| 4. | DB03147 | FAD | 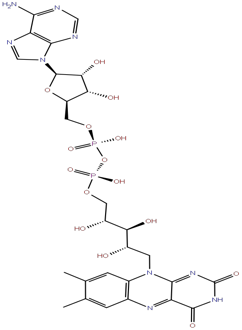 | -8.51 | -55.834 | Thr100, Asp102, Asn103, His267, Arg283 | Lys156, Lys265, His267 | His267 |
| 5. | DB09156 | Iopromide | 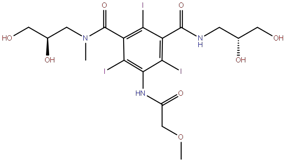 | -6.995 | -42.571 | Thr100, Asn103, Asp280, Asp282, Arg283 | - | - |
| 6. | DB01698 | Rutin | 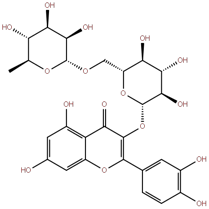 | -5.707 | -49.447 | Thr100, Asn103, Asp280, Asp282 | Arg283 | Thr100  His267 (Pi-stacking), Arg283 (Pi-stacking) |
| 7. | DB012434 | Steviolbioside | 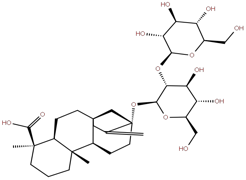 | -6.145 | -40.299 | Thr100, Asn103, Lys153, His267,  Arg283 | - | Ala86 |
| 8. | DB012942 | Lactitol | 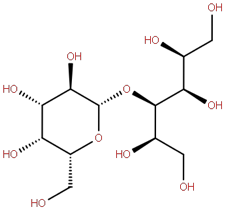 | -7.198 | -37.693 | Thr100, Asp102, Asn103, Lys156, Asp280, Arg283 | His267 | - |
| 9. | DB01601 | Lopinavir | 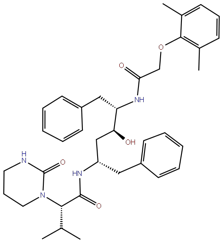 | -3.293 | -40.426 | Glu88, Thr100, His267, Asp282, Arg283 | - | Thr100, Asp282, His267 (Pi-stacking) |
| 10. | DB00358 | Mefloquine | 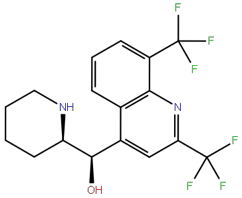 | -3.332 | -21.168 | Asp102 | - | - |
| 11. | DB01264 | Darunavir | 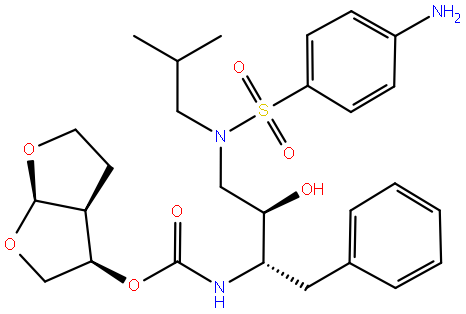 | -2.319 | -30.686 | Thr100, His267, Asp280, Arg283 | - | His267 |
| 12. | DB00503 | Ritonavir | 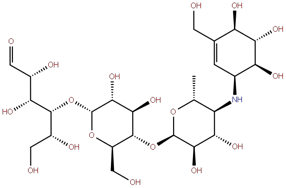 | -2.716 | -43.946 | Thr100, Asp102, Asp282, Arg283 | - | Lys99, His267(Pi-stacking & Pi-cation), Arg283(Pi-Stacking) |

**Supplementary Information Table 5:** Compounds used in the study, their cytotoxicity, antiviral activity and the virus loads in infected and treated MRC-5 cells

| **S.No.** | **Compound** | **Assay Conc. (Range [µM])** | **% Cytotoxicity (Range)** | **CC_50_ (µM)** | **Statistical Significance** | **% Antiviral Activity (Range)** | **EC50** | **Selectivity Index**  **(CC_50_/EC_50_)** | **Statistical Significance** | **% Reduction in Viral Load (Range)** | **Log_10_ Reduction in Viral Load (Range)** | **Statistical Significance** |
| --- | --- | --- | --- | --- | --- | --- | --- | --- | --- | --- | --- | --- |
| 1. | Favipiravir | 15.6-1000 | -8.1 to +67.5 | 665 | R = -0.9375  *p* = 0.0018 | -0.6 to +26.9 | N.D. | N.A. | R = 0.7933  *p* = 0.0333 | 0.0 to +81.6 | +0.74 to 0.0 | R = -0.9257  *p* = 0.0028 |
| 2. | Acarbose | 121.1 - 7750 | -15 to +7 | >7750 | R = -0.4697  *p* = 0.2876 | +0.3 to +7.2 | >7750 | N.D. | R = 0.7499  *p* = 0.0523 | N.D. | N.D. | N.A. |
| 3. | Darunavir | 11.4 - 730 | -14.4 to +97.2 | 302.95 | R = -0.9362  *p* = 0.0019 | -6.9 to +34.7 | N.D. | N.A. | R = 0.8111  *p* = 0.0498 | 0.0 to +97.6 | 0.0 to +1.62 | R = -0.8192  *p* = 0.0461 |
| 4. | FAD.H_2_O.2Na | 18.8 - 1200 | -10.5 to +12.7 | >1200 | R = -0.7874  *p* = 0.0356 | -1.3 to +8.1 | >1200 | N.D. | R = 0.6685  *p* = 0.1007 | N.D. | N.D. | N.A. |
| 5. | GRL0617 | 11.4 - 730 | +6.2 to +76 | 137.4 | R = -0.994  *p* < 0.0001 | -1.9 to +33.7 | N.D. | N.A. | R = 0.7661  *p* = 0.0757 | Inconsistent | Inconsistent | R = -0.0712  *p* = 0.8845 |
| 6. | Lactitol | 45.3 - 2900 | -8.9 to +1.8 | >2900 | R = -0.2902  *p* = 0.5278 | -1.2 to +5.3 | >2900 | N.D. | R = 0.8179  *p* = 0.0246 | N.D. | N.D. | N.A. |
| 7. | Lopinavir | 1.3 - 80 | -9.6 to +99.9 | 67 | R = -0.6567  *p* = 0.1091 | -6.3 to +72.4 | 33 | 2.03 | R = 0.8482  *p* = 0.0328 | +96.2 to +29.1 | +1.42 to +0.15 | R = -0.8629  *p* = 0.0269 |
| 8. | Mefloquine-HCl | 3.8 - 240 | +11.5 to +93.2 | 172.8 | R = -7835  *p* = 0.0371 | -0.4 to +60.6 | 108.3 | 1.6 | R = 0.7949  *p* = 0.0488 | +84.7 to +38.4 | +0.81 to +0.21 | R = -0.7659  *p* = 0.0758 |
| 9. | Oxidized L-Glutathione | 23.4 - 1500 | -17.5 to -3.9 | >1500 | R = -0.2694  *p* = 0.559 | -2.2 to +6.7 | >1500 | N.D. | R = 0.8765  *p* = 0.0096 | N.D. | N.D. | N.A. |
| 10. | Ritonavir | 4.3 - 278 | +20.6 to +100.1 | 29.4 | R = -0.9784  *p* = 1.3E-4 | -3.8 to +36.4 | N.D. | N.A. | R = 0.9138  *p* = 0.03 | +73.3 to -625.5 | +0.57 to -0.86 | R = -0.9476  *p* = 0.0012 |
| 11. | Rutin.3H_2_O | 9.4 - 600 | -5.9 to +89.5 | 351 | R = -0.8545  *p* = 0.0143 | -1.1 to +37.1 | N.D. | N.A. | R = 0.7849  *p* = 0.0644 | +38.4 to -455.4 | +0.21 to -0.74 | R = -0.9452  *p* = 0.0044 |

**Supplementary Information Table 6:** Effects of the drugs on the SARS-CoV-2 cell viability and replication in live cells

| **S. No** | **Compound Name** | **Concentration** | **% Cell Viability** | **% Inhibition** |
| --- | --- | --- | --- | --- |
| 1. | Remdesivir | 10 µM | 99.23 | 99.2 |
| 2. | Mefloquine | 5 µM | 45.6 | NA |
|  |  | 0.5 µM | 93.2 | 30.6 |
|  |  | 0.25 µM | 93.7 | NA |
